# Robust pan-junctional reinforcement preserves the gut epithelial barrier under mechanical stress

**DOI:** 10.1126/sciadv.aed3122

**Published:** 2026-10-30

**Authors:** Vishnu Krishnakumar, Amalia Ferran, Sandra Bernat-Fabre, Justine Creff, Silvia Kocanova, Nicolas Cenac, Denis Krndija

**Affiliations:** ^1^ University of Toulouse & CNRS, Centre de Biologie Intégrative (CBI)–UMR5077, Toulouse, France.; ^2^ Institut de Recherche en Santé Digestive (IRSD)–INSERM U1220, Toulouse, France.

## Abstract

Epithelia are specialized tissue barriers that safeguard the organism’s internal milieu from the external environment, a function critically dependent on intercellular junctions. In the colon, this barrier is repeatedly challenged by mechanical distension from feces, but it is unknown whether and how the colon adapts to this stress, which could otherwise compromise barrier integrity. Here, we show that feces-mediated mechanical distension triggers coordinated remodeling at cell and tissue scales, consistent with a mechanoadaptative response. This response includes recruitment of junctional proteins at all three types of adhesive cell-cell junctions (tight junctions, adherens junctions, and desmosomes). We identified protein-specific temporal profiles of junctional enrichment during distension, with ZO-1, E-cadherin, desmocollin 2, and plakoglobin exhibiting sustained junctional enrichment, whereas desmoplakin and keratin 8 showed progressive accumulation peaking at 15 min of distension. Distension was also accompanied by perijunctional recruitment and activation of nonmuscle myosin II (NMMII). Through genetic, pharmacological, and mechanical perturbations, we demonstrated that NMMII activation is required for epithelial mechanoadaptation. This process depends on extracellular calcium availability and mechanosensitive ion channel activity, supporting a role for calcium-dependent mechanosensitive signaling in NMMII-mediated junctional reinforcement. Loss of NMMII function impaired the junctional response to distension across all three junctional complexes, including desmosomes, resulting in disorganized junctions and barrier breach. Together, our findings uncover a robust physiological mechano-adaptive response of the adult colonic epithelium to an extrinsic mechanical stress, whereby coordinated reinforcement of all junctional complexes, requiring myosin II activity and calcium-dependent mechanosensitive signaling, preserves intestinal barrier integrity.

## INTRODUCTION

Epithelia are the specialized tissue barriers that protect the internal environment of an organism from the hostile external environment, thereby ensuring normal tissue function and homeostasis. The gut epithelium must withstand multiple environmental insults, including diverse microbiota, toxins, and mechanical forces (e.g., compression and stretch) arising from distension by luminal contents and muscle-driven peristalsis (*1*–*3*). Despite these challenges, the gut epithelium remarkably maintains its critical roles in nutrient and water absorption while preserving barrier integrity throughout life. The adult colon exhibits a distinct architecture in which the mucosa—comprising the epithelium and underlying lamina propria—folds into the lumen and is organized into two main regions: crypts, which contain proliferative stem and progenitor cells, and plateaus, the intercryptal regions lined with differentiated epithelial cells directly exposed to luminal contents. However, how this mucosal architecture physically adapts to the extrinsic mechanical load imposed by feces in vivo—and, in particular, how the colonic epithelium preserves barrier integrity to maintain homeostasis and prevent pathogen translocation—remains poorly understood. Epithelial tissue integrity is maintained by adhesive cell-cell contacts, comprising tight junctions (TJs), adherens junctions (AJs), and desmosomes. TJs form the most apical junctional complex, physically sealing the paracellular space through the transmembrane proteins occludin and claudins, and play key roles in regulating apico-basal polarity and selective ion permeability (*4*). They are mechanically linked to the underlying actomyosin belt via the zonula occludens (ZO) family of scaffolding proteins (*5*). In particular, ZO-1 responds to intrinsic mechanical forces arising during cellular processes through recruitment to the junctions (*6*, *7*). AJs are located beneath the TJs and mechanically integrate epithelial cells through coupling of cadherins to perijunctional actomyosin belts via catenins and cytoplasmic linkers. Studies in simple epithelia and developmental models have shown that AJ proteins respond to mechanical tension by clustering, undergoing conformational changes, and engaging the contractile actomyosin network, thereby reinforcing junctional complexes to maintain tissue cohesion (*8*–*11*). Desmosomes, positioned basolaterally beneath AJs, provide mechanical stability by anchoring to intermediate filaments (*12*–*14*). In contrast to TJs and AJs, however, very little is known about whether desmosomes can sense or adapt to extrinsic forces in vivo, or how they might interact with the actomyosin cytoskeleton. This gap in knowledge is especially notable given their central role in mechanical resilience.

During morphogenesis, epithelial cells use multiple strategies to sense mechanical forces, including adhesion complexes and mechanosensitive ion channels, which may trigger junctional remodeling to preserve tissue integrity (*6*, *7*). Yet, in adult epithelia in vivo, it remains poorly understood how extrinsic forces are sensed and how adhesive complexes—particularly desmosomes—and their coupling to actomyosin and intermediate filaments contribute to tissue integrity and barrier maintenance.

Here, we investigated how the colonic epithelium responds to physiological mechanical forces by combining high resolution three-dimensional (3D) tissue imaging with mechanical and pharmacological perturbations in in vivo (mouse models), ex vivo (gut explant), and in vitro (2D gut organoid) systems. We found that TJs, AJs, and desmosomes respond to extrinsic mechanical force through recruitment of their respective proteins, with distinct kinetics. Desmosomes, in particular, displayed progressive reinforcement during distension, highlighting their adaptive mechanoresponse in the gut. Non-muscle myosin II emerged as a critical mediator of this junctional recruitment under mechanical stress—its inhibition or depletion impaired force-induced recruitment across all the junctional complexes and led to barrier defects. Finally, our data suggest that mechanosensitive ion-channel activity and extracellular calcium availability contribute to this response, converging on myosin II activation to promote coordinated junctional reinforcement.

## RESULTS

### Feces-mediated distension of colon leads to cell- and tissue-scale epithelial remodeling and junctional recruitment

To investigate the impact of mechanical forces in the adult murine colon, we first assessed the organ’s gross morphology with respect to the presence of fecal pellets. Regions containing feces, interspersed with feces-free regions, appeared visibly distended (Fig. 1A). By confocal imaging of colonic thick sections, either distended or nondistended by feces, we observed unfolding of mucosal folds specifically in the distended regions (Fig. 1B and fig. S1A). Distension was associated with tissue-level morphological changes in crypt and plateau organization: The intercryptal plateaus were expanded, whereas the crypts appeared shorter (Fig. 1, B and D). Since the plateau regions are in direct contact with the gut lumen and feces—and thus more exposed to luminal contents and microbiota—we focused our detailed morphometric analyses on these regions. Epithelial cell aspect ratio was significantly altered (34% decrease) (Fig. 1, C and E), suggesting that colonic epithelial cells undergo substantial deformation and associated strain upon fecal distension. To understand how the epithelial cells respond to the distension-induced strain, we assessed the adhesive cell-cell junctions (TJs, AJs, and desmosomes) by immunofluorescence on thick sections with luminal side facing up (en face), enabling high-resolution 3D imaging of the cell-cell junctions. TJ proteins (ZO-1 and occludin), along with AJ protein E-cadherin (E-cad) were enriched at the junctions in feces-distended regions (2.4-, 2.4-, and 1.8-fold, respectively) compared to the adjacent, nondistended regions (Fig. 1, F, I, and J, and fig. S1, A to C).

**Fig. 1.**
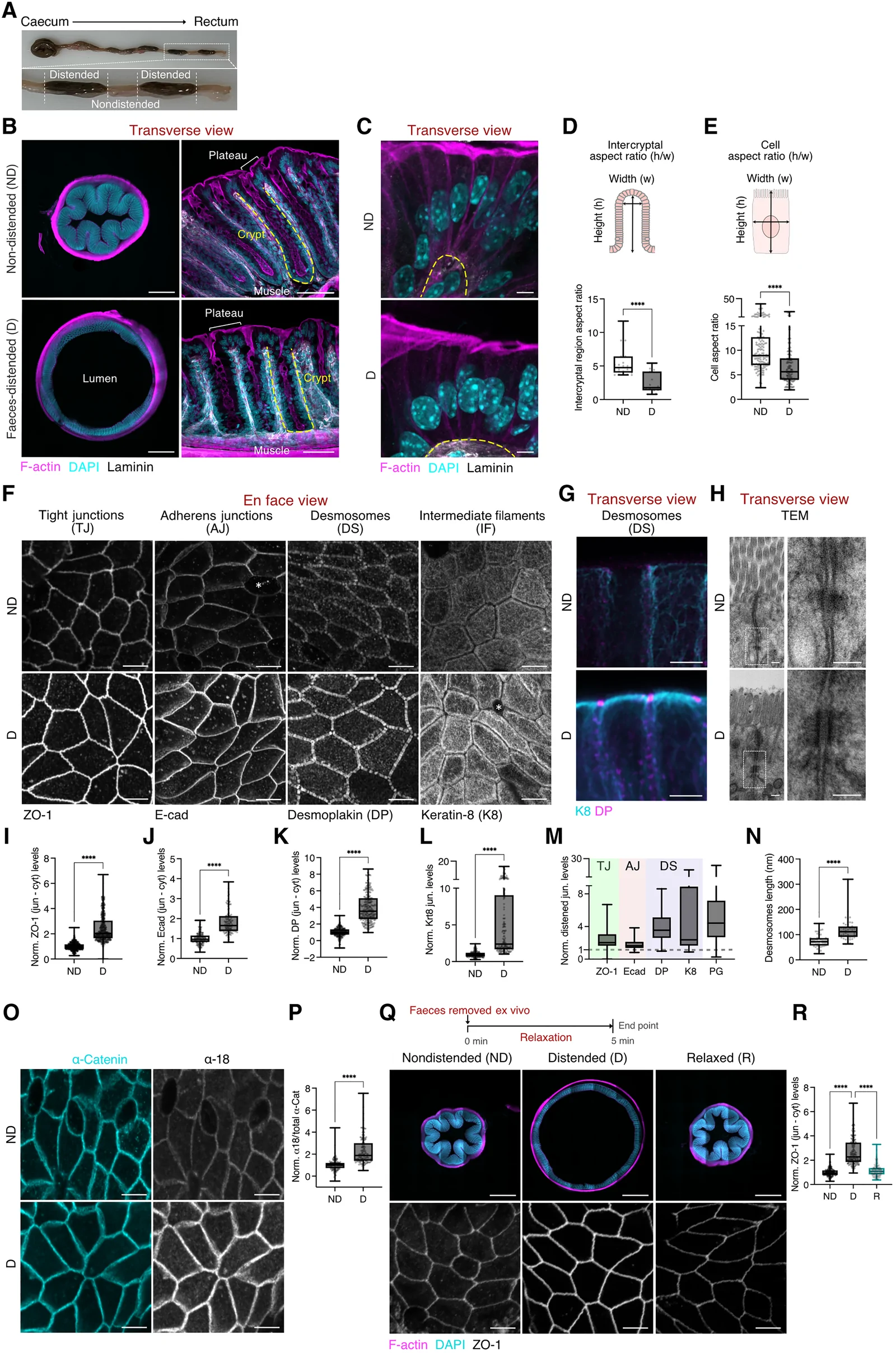
Feces-mediated colonic distension leads to tissue remodeling and junctional recruitment. (**A**) Mouse colon showing nondistended (ND) and feces-distended regions (D). (**B**) Transverse mouse colon section with lumen, plateau regions (white brackets), muscle layers, and crypts (yellow dashed outlines) indicated; F-actin, 4′,6-diamidino-2-phenylindole (DAPI), and laminin. Left: Tiled confocal reconstruction. Scale bars, 500 μm (left) and 50 μm (right). (**C**) Transverse images of plateau regions; basal cell side outlined with yellow dashed line; F-actin, DAPI, and laminin. Maximum intensity Z projections (MIP; 2 to 4 μm range). Scale bars, 5 μm. (**D**) Intercryptal aspect ratio measurements (top) and quantification (bottom); *n* = 5 to 9 intercryptal regions per condition, *N* = 4. (**E**) Cell aspect ratio measurements (top) and quantification (bottom); *n* > 28 cells per condition, *N* = 4. (**F**) En face images showing ZO-1, E-cad, DP, and K8. DP marks the apical-most desmosomes. MIP (1 to 3 μm). Scale bars, 5 μm. (**G**) Transverse images of K8 and DP. MIP (2 to 4 μm). Scale bars, 5 μm. (**H**) Transverse TEM images showing apical-most desmosomes (white box; enlarged insets on the right). Scale bars, 100 nm. (**I** to **L**) Junctional intensity for ZO-1 (I), E-cad (J), DP (K), and K8 (L) levels; *n* = 34 to 85 junctions per condition; *N* = 4 to 9. (**M**) Fold changes in junctional protein levels in D regions. Dashed line indicates the normalized ND average. (**N**) Desmosome length quantification; *n* = 7 to 27 desmosomes/condition; *N* = 3. (**O**) En face images showing α-catenin and α-18. MIP (1- to 3-μm range). Scale bars, 5 μm. (**P**) Ratio of junctional intensity (α-18/α-catenin; *n* = 48 to 60 junctions/condition; *N* = 3). (**Q**) Relaxation assay approach (top). Stitched transverse confocal images showing ND, D, and relaxed (R) regions; F-actin, and DAPI (middle). Scale bars, 500 μm. En face ZO-1 images (bottom). MIP (1 to 3 μm). Scale bars, 5 μm. (**R**) Junctional ZO-1 intensity; *n* = 60 to 80 junctions/condition; *N* = 3. Statistical analyses: Mann-Whitney *U* test [(D), (E), (I) to (L), (N), and (P)] or Kruskal-Wallis followed by Dunn’s multiple comparison (R). *: goblet cell.

Desmosome-associated proteins [desmocollin 2 (DSC2), plakoglobin (PG), desmoplakin (DP), and keratin 8 (K8)] were also enriched at the junctions (Fig. 1, F, K, and L, and fig. S1, D and E) with DP, PG, and K8 showing even higher recruitment (3.9-, 5.3-, and 4.8-fold, respectively) upon distension when compared to the TJ and AJ proteins (Fig. 1M).

Desmosomal adaptation upon distension was also evident in en face views (Fig. 1F) and in transverse colonic sections (Fig. 1G), where DP and K8 showed perijunctional and broader apical enrichment. Because desmosomes are punctate, discontinuous adhesive plaques distributed below the AJ level and along the lateral membrane, we focused our quantitative analyses on the apical-most desmosomes, defined as the desmosomal plaques positioned immediately below the AJ/F-actin plane. This apical-most population could be consistently resolved by both confocal microscopy and transmission electron microscopy (TEM). At this level, distension induced a significant increase in desmosomal plaque length, as measured by TEM (nondistended: 74 nm; distended: 117 nm; Fig. 1, H and N). In addition, en face confocal sections acquired in deeper optical planes showed that more basolateral desmosomes, located below the apical-most desmosomal plane, also displayed increased DSC2, DP, and K8 enrichment in feces-distended regions (fig. S1, H and I).

In addition to plaque elongation and apical desmosomal enrichment, distension was associated with a redistribution of desmosomal components from nonjunctional pools toward cell-cell junctions. This was most apparent for PG, which showed a marked reduction in cytoplasmic staining together with increased junctional accumulation in feces-distended regions (fig. S2, A to C). DP showed a similar trend toward junctional enrichment, although the reduction in nonjunctional signal was less pronounced (fig. S2, D and E).

We did not observe any difference in total protein levels of key junctional markers between feces-distended and nondistended regions, suggesting a force-dependent relocalization of junctional proteins rather than de novo synthesis (fig. S2, F and G).

To further characterize the epithelial response to feces-mediated distension, we examined additional markers of junctional mechanotransduction and actomyosin contractility. Distended regions showed enhanced α-18 staining, reporting force-dependent α-catenin unfolding, as well as increased phosphorylation of myosin light chain at serine-19 (pMLC S19), indicating elevated myosin II activity (Fig. 1, O and P, and fig. S1, F and G). In contrast, β-catenin, a core AJ protein, did not show junctional recruitment upon distension, suggesting selective force-induced remodeling rather than general accumulation of AJ components (fig. S2H). The enrichment of junctional proteins at cell-cell contacts, together with comparable total protein levels in distended and nondistended regions, strongly suggests that this response arises from force-dependent redistribution of pre-existing proteins rather than de novo synthesis, as previously shown for E-cad, which can be delivered through vesicle trafficking or lateral diffusion and clustering within the membrane (*10*, *15*–*17*). Similarly, PG displayed diffuse cytoplasmic localisation in nondistended tissue; upon tissue distension, it was cleared from the cytoplasm and enriched at junctions (fig. S2, A to C), resembling the force-mediated relocalization of ZO-1, previously attributed to phase separation under mechanical force (*6*).

To explore the stability of the accumulated junctional complexes and decipher whether the distension is required to maintain the recruited proteins at the junctions, we analyzed gut fragments with/without feces and upon feces removal ex vivo.

Upon feces removal, mucosal folds rapidly reformed and junctional enrichment of ZO-1, PG, and DP was lost within 5 min (Fig. 1, Q and R, and fig. S2, I to N), indicating that junctional reinforcement is rapidly reversible upon tissue relaxation. To further explore whether junctional DP recruitment reflects regulated desmosomal remodeling, we analyzed DP phosphorylation (pDP) at Ser^2849^, a modification associated with reduced DP-intermediate filament locking and increased desmosomal protein exchange (*18*, *19*). The quantification of junctional pDP relative to total junctional DP revealed increased Ser^2849^ phosphorylation in distended regions (fig. S2, O and P), suggesting that distension-associated DP recruitment involves dynamic regulation of DP-keratin coupling rather than static protein accumulation.

Together, these data suggest that the colon responds to extrinsic physiological mechanical force through multiscale adaptation, including tissue- and cell-level epithelial remodeling and junctional recruitment of adhesive TJ, AJ, and desmosomal proteins.

### Time-resolved colonic distension in vivo reveals differential junctional recruitment kinetics and transient apical constriction

The duration of fecal residence and transit through the mouse colon is not well characterized and may depend on multiple factors, including stress levels, diet, and microbiota (*20*, *21*). In addition, fecal pellets are variable in size (*22*), which we also observed in our measurements of pellet size (fig. S2, Q and R). To achieve controlled distension with precise temporal resolution and consistent volume, we introduced an inflatable catheter into the distal colon of anesthetised mice (Fig. 2A) (*23*). To investigate the recruitment kinetics of junctional complexes, colonic distension was performed at a constant inflation volume and maintained for 5, 15, or 30 min. These intervals were selected to cover short to more sustained stimulation, as the residence time of fecal pellet at a given site is difficult to determine directly. Estimates of mouse colonic transit are around 2 hours (*24*), but this likely varies with fecal viscosity and consistency (*25*), suggesting that fecal distension can exert pressure on the colonic wall over extended periods. Catheter-mediated distension induced alterations in cell aspect ratio and intercryptal morphology that were comparable to those observed with feces-mediated distension (fig. S3, A and B). Thus, this approach provides a controlled, time-resolved fixed-tissue assay to assess junctional organization during acute mechanical distension. Consistent with feces-mediated distension, ZO-1, E-cad, PG, and DSC2 showed significant junctional recruitment upon catheter-mediated distension, which was already evident from 5 min, the earliest time point analyzed, and remained broadly sustained up to 30 min (Fig. 2, B, C, and E, and fig. S3, C and D).

**Fig. 2.**
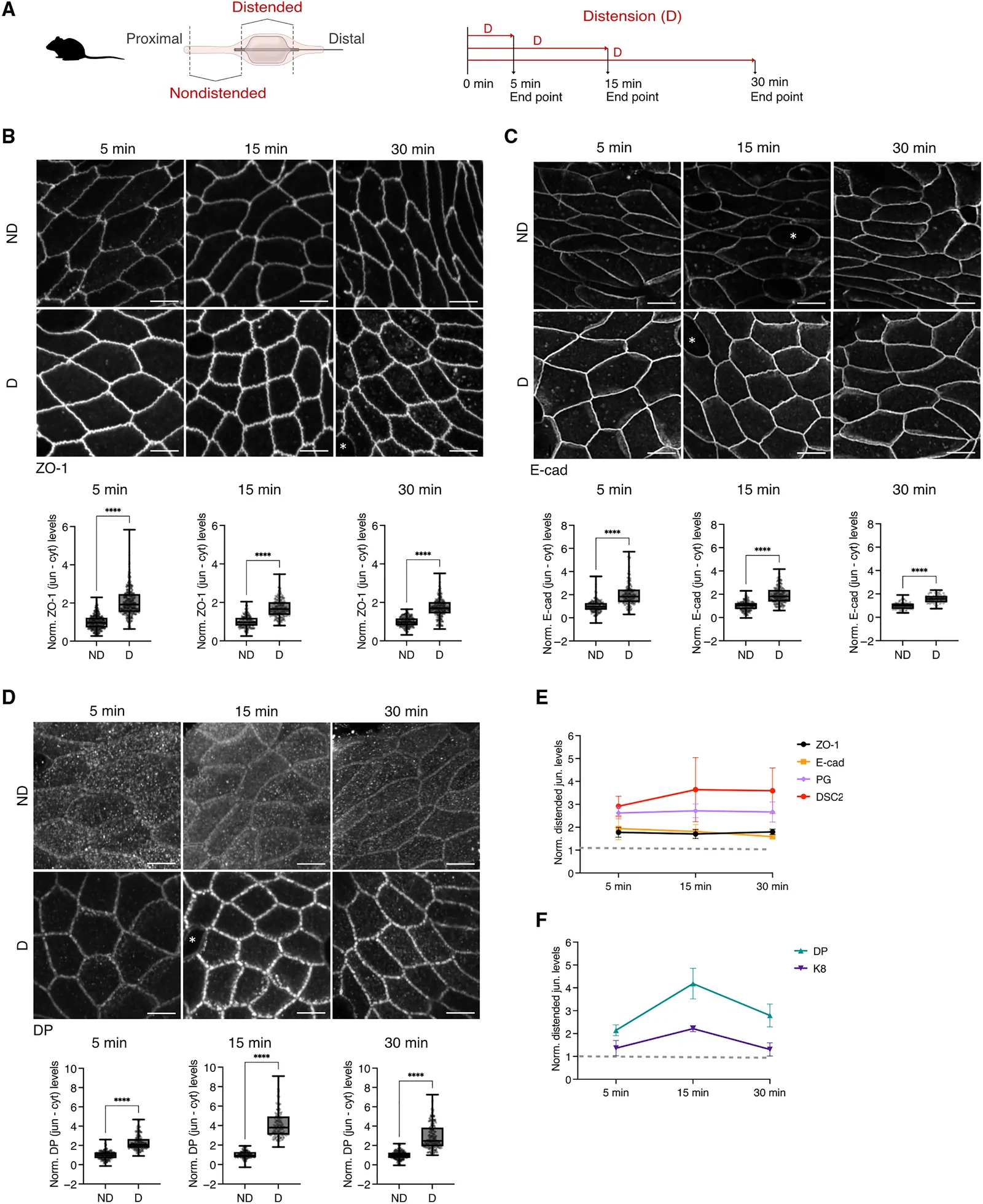
Time-resolved distension reveals distinct junctional recruitment dynamics. (**A**) Scheme showing experimental approach for catheter-mediated in vivo distension of the mouse colon. (**B**) Top: En face images of colonic epithelium stained for ZO-1 in ND and catheter-mediated distended (D) regions after 5, 15, and 30 min of distension. MIP (1- to 3-μm range). Scale bar, 5 μm. Bottom (left to right): Junctional ZO-1 intensity quantification after 5, 15, and 30 min of distension; *n* = 26 to 125 junctions/condition; *N* = 4 to 7. (**C**) Top: En face images of colonic epithelium, stained for E-cad in ND and D regions after 5,15 and 30 min of distension. MIP (1- to 3-μm range). Scale bar, 5 μm. Bottom (left to right): Junctional E-cad intensity quantification after 5, 15, and 30 min of distension; *n* = 23 to 64 junctions/condition; *N* = 2 to 4. (**D**) Top: En face images of colonic epithelium stained for DP in ND and D regions after 5, 15, and 30 min of distension. MIP (1- to 3-μm range). Scale bar, 5 μm. Bottom (left to right): Junctional DP intensity quantification after 5, 15, and 30 min of distension; *n* = 21 to 65 junctions/condition; *N* = 3 to 6. (**E**) Comparative line plot showing junctional intensity fold change for ZO-1, E-cad, DSC2 and PG after 5, 15 and 30 min of distension. Dotted line represents the normalized average for ND. (**F**) Comparative line plot showing junctional intensity fold change for DP and KRT8 after 5, 15, and 30 min of distension. Dotted line represents the normalized average for ND. Statistical analysis was performed using the Mann-Whitney *U* test. *: goblet cell.

In contrast, DP and K8 displayed a more dynamic temporal profile. Both proteins increased between 5 and 15 min, with DP rising from 2.1-fold to 4.1-fold and K8 from 1.3-fold to 2.2-fold, followed by partial attenuation at 30 min (DP: 1.5-fold; K8: 1.7-fold) (Fig. 2, D and F, and fig. S3E). Consistent with the DP/K8 kinetics observed by immunofluorescence, TEM analysis revealed a similar temporal pattern in desmosomal organization, with plaque length remaining unchanged at 5 min, increasing at 15 min, and partially decreasing at 30 min while remaining above baseline (fig. S3F).

Together, these data reveal protein-specific temporal patterns of junctional enrichment upon acute mechanical distension of the colonic epithelium. While ZO-1, E-cad, PG, and DSC2 were rapidly recruited and broadly sustained, DP and K8 displayed delayed peak enrichment followed by partial attenuation. This suggests that desmosomal reinforcement involves temporally distinct layers of regulation, with sustained recruitment of core adhesive components and more dynamic remodeling of the DP-keratin linkage.

### Perijunctional myosin II accumulates during distension and is required for junctional remodeling in vivo

Upon controlled distension, we observed a transient reduction in apical cell area (22%) (fig. S4, A and B). Since apical constriction is typically driven by actomyosin contractility, this suggests activation of myosin II in response to colonic distension. We thus decided to investigate the potential role of nonmuscle myosin II (NMMII), a key cytoskeletal motor involved in epithelial remodeling under force. NMMIIA, the predominant nonmuscle myosin II isoform localized at the perijunctional actomyosin belts, is well characterized for its role in generating contractile forces (*26*, *27*). To examine the kinetics of perijunctional NMMIIA in distended versus nondistended tissues, we performed catheter-mediated in vivo distension, as before, in mice expressing green fluorescent protein (GFP)tagged endogenous NMMIIA (*26*). NMMIIA showed pronounced enrichment at junctions and across the apical cortex during catheter-mediated distension, peaking at 15 min (2.6-fold increase compared to 5 min) and coinciding with the transient reduction in apical cell area (Fig. 3A and fig. S4C). We also confirmed that feces-mediated distension induced NMMIIA recruitment to the perijunctional belt (fig. S4, D and E). To assess whether apical/perijunctional NMMIIA accumulation was associated with actomyosin activation, we analyzed pMLC S19 staining across the catheter-mediated distension time course. pMLC showed increased apical/perijunctional enrichment during distension that paralleled the NMMIIA-GFP response (Fig. 3B), supporting the activation of NMII-dependent junctional contractility during acute mechanical distension. To investigate the role of NMMIIA in epithelial junctional remodeling in response to distension, we used an inducible and gut epithelium-specific NMMIIA knockout (NMMIIA-KO) mouse model (Fig. 3C), which is indistinguishable from controls in terms of body weight and fecal size and morphology (fig. S4F). At the tissue scale, epithelial NMMIIA depletion did not prevent feces-associated mucosal deformation: NMMIIA-KO colons retained folded architecture in nondistended regions and underwent unfolding/deformation in feces-distended regions, with distension-induced changes in intercryptal aspect ratio comparable to those observed in control tissue (fig. S4, G to I). Upon NMMIIA depletion, we observed a significant reduction in the fecal distension-dependent recruitment of multiple markers across all three junctional systems, including TJ, AJ, and desmosomal components together with associated K8 filaments (Fig. 3, C to F, and figs. S4, J to L and O to R and S5A). Reduced ZO-1 recruitment was accompanied by irregular and discontinuous ZO-1 staining in distended NMMIIA-KO regions (Fig. 3C and fig. S4N), suggesting that impaired NMMIIA-dependent reinforcement is associated with altered TJ organization under mechanical stress. TEM analysis further showed that apical desmosomal plaque length increased significantly upon distension in control tissue, whereas this increase was not detected in NMMIIA-KO tissue (Fig. 3, G and H), supporting impaired desmosomal reinforcement upon loss of NMMIIA. In parallel, the apical cell area, already elevated in nondistended NMMIIA-KO tissue, increased further upon distension, exceeding the levels observed in controls (fig. S4M), indicating that loss of NMMIIA removes contractile restraint, allowing greater epithelial deformation under mechanical load. We observed no reduction in the total levels of junctional proteins in NMMIIA-KO mice compared to controls, indicating that NMMIIA promotes junctional complex accumulation through recruitment rather than by regulating their expression (fig. S5, B and C). Despite being significantly reduced, junctional recruitment in response to distension was not fully abolished in NMMIIA-KO. We thus investigated whether other myosin II isoforms could compensate. Of the other two isoforms—NMMIIB and NMMIIC—NMMIIB is not detectably expressed in the gut epithelium (*27*), while NMMIIC localizes to the terminal web, a cortical actin-rich zone distinct from the perijunctional actomyosin belts (*28*). Upon distension in wild-type mice, NMMIIC was also recruited to the perijunctional belt (fig. S5, D and E). However, unlike NMMIIA, for which levels peaked at 15 min, NMMIIC levels remained broadly sustained across the analyzed time points. In NMMIIA-KO mice, NMMIIC was already detectable at junctions under nondistended conditions and increased further upon distension (Fig. 3, I and J). Total NMMIIC protein levels were unchanged in NMMIIA-KO mice (fig. S5, B and C), consistent with redistribution of the existing NMMIIC pool rather than increased protein abundance. To assess the extent of actomyosin activation in the absence of NMMIIA, we analyzed pMLC. NMMIIA-KO tissue still showed significant perijunctional pMLC enrichment upon distension, although at substantially lower levels than control tissue (fig. S5, F and G). Since pMLC is not isoform-specific, these data do not demonstrate direct functional compensation by NMMIIC. However, they indicate that residual actomyosin activation persists in the NMMIIA-KO epithelium and suggest that NMMIIC recruitment may contribute to the remaining junctional response. Together, our data show that myosin II is recruited to perijunctional regions during distension, accompanied by increased actomyosin activation and that NMMIIA is required for robust junctional remodeling in response to mechanical stress in vivo.

**Fig. 3.**
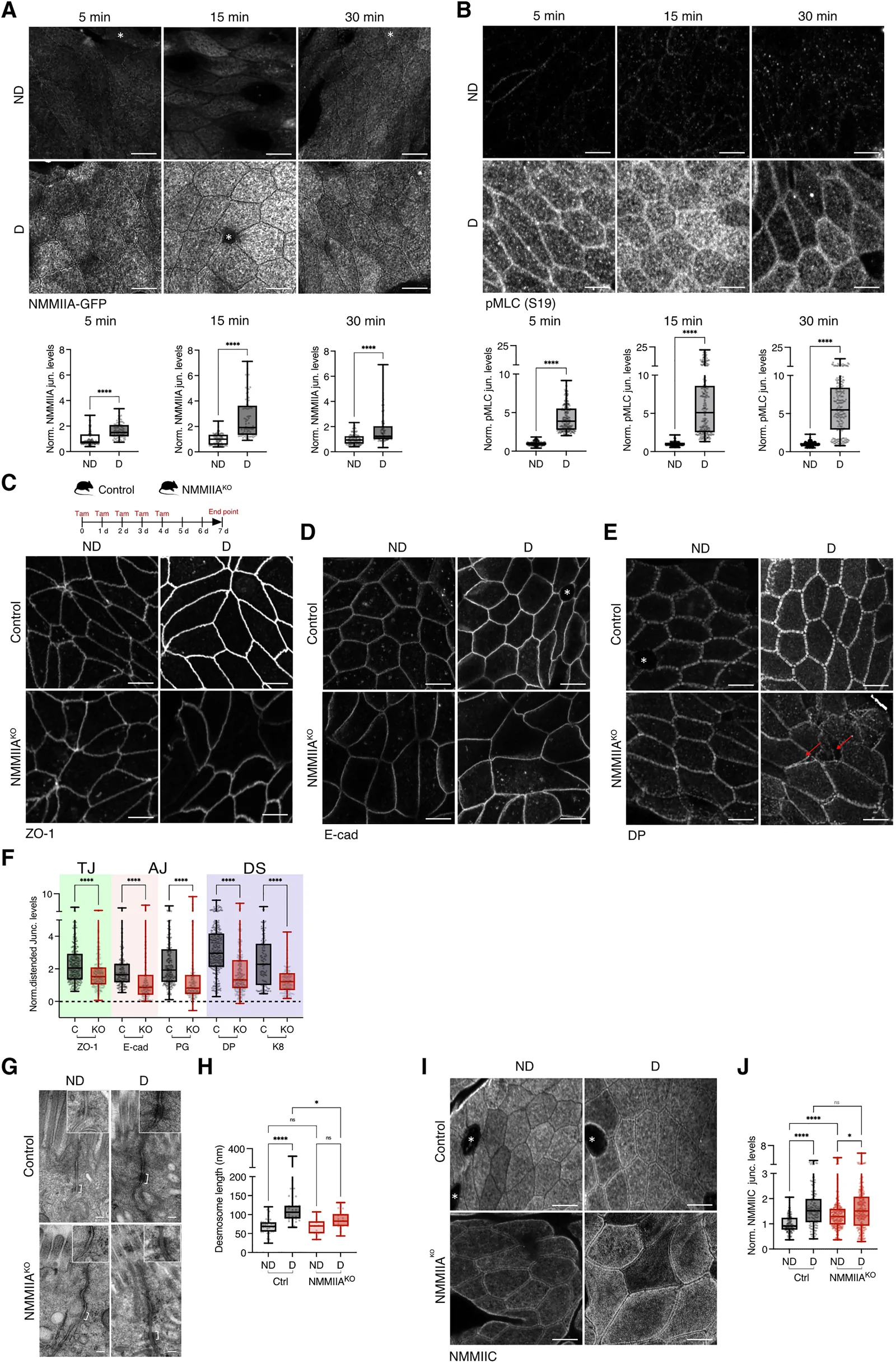
Junctional remodeling during colonic distension requires nonmuscle myosin IIA. (**A**) Top: En face images of colonic epithelium showing NMMIIA-GFP in ND and catheter-mediated distended (D) regions after 5, 15, and 30 min. MIP (1- to 3-μm range). Scale bars, 5 μm. Bottom (left to right): Junctional NMMIIA-GFP intensity after 5, 15, and 30 min; *n* = 35 to 62 junctions/condition; *N* = 2. (**B**) Top: En face images of colonic epithelium showing pMLC (S19) in ND and catheter-mediated D regions after 5, 15, and 30 min. MIP (1- to 3-μm range). Scale bars, 5 μm. Bottom (left to right): Junctional pMLC (S19) intensity after 5, 15, and 30 min; *n* = 49 to 74 junctions/condition; *N* = 3. (**C**) Top: Scheme of the inducible NMMIIA knockout strategy. Control: Villin-CreERT2^−/−^; NMMIIA^KO^: Villin-CreERT2^+/−^. Bottom: ZO-1 images from control and NMMIIA^KO^ mice from ND and feces-D regions. MIP (1- to 3-μm range). Scale bars, 5 μm. (**D** and **E**) En face images stained for E-cad (D) and DP (E) in control and NMMIIA^KO^ mice from ND and feces-D regions. MIP (1- to 3-μm range). Red arrow indicates fractured DP junctions. Scale bars, 5 μm. (**F**) Fold changes in junctional protein levels in feces-D regions of control and NMMIIA^KO^ mice. Dotted line indicates the normalized ND average. (**G**) TEM images showing desmosomes (white bracket; insets) in control and NMMIIA^KO^ mice from ND and feces-D regions. Scale bars, 100 nm. (**H**) Desmosome length quantification in control and NMMIIA^KO^ mice from ND and feces-D regions; *n* = 10 to 27 junctions/condition, *N* = 2 (**I**) En face images stained for NMMIIC in control and NMMIIA^KO^ mice from ND and feces-D regions. MIP (1- to 3-μm range). Scale bars, 5 μm. (**J**) Junctional NMMIIC intensity quantification in ND and D regions of control and NMMIIA^KO^ mice; *n* = 30 to 60 junctions/condition, *N* = 4. Statistical analyses: Mann-Whitney *U* test [(A) and (B)] and Kruskal-Wallis test followed by Dunn’s multiple comparison [(F), (H), and (J)]. *: goblet cell.

### Junctional recruitment in response to mechanical distension depends on myosin II activation mediated by mechanosensitive ion channels

To further explore the mechanism of force-mediated junctional recruitment, we turned to pharmacological manipulation (Fig. 4A) and adapted our ex vivo gut explant culture system (*29*) to colon tissue. Explants from nondistended regions of the colon were maintained at an air-liquid interface and subjected to defined compression from above using a fixed weight to mimic colonic distension (Fig. 4B and fig. S6, A and B). Recapitulating the in vivo system, the distension of control explants induced robust accumulation of junctional complexes and, importantly, triggered phosphorylation of the myosin regulatory light chain (MLC), marking the activation of contractility under mechanical stress (Fig. 4, D and F). To exclude that this response could be due to smooth muscle contractility in the explant, we have also optimized and performed colonic organoid monolayer culture in 2D on Transwell inserts (*30*); using the same approach with a fixed weight, we observed junctional recruitment and phosphorylation of MLC (fig. S6, C to F), suggesting that this response does not depend on the muscle layer.

**Fig. 4.**
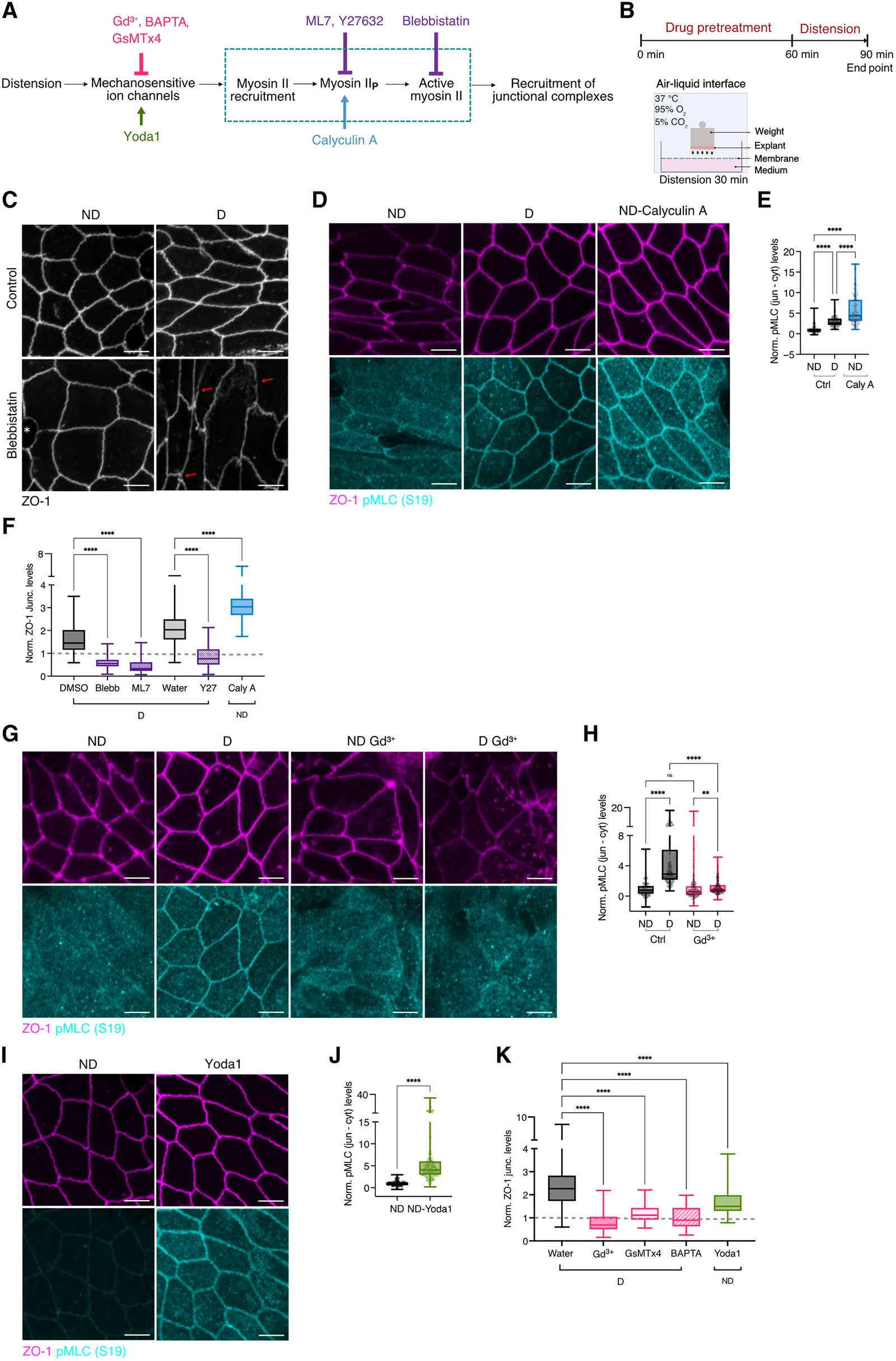
Myosin II activity regulated by mechanosensitive calcium influx is essential for junctional recruitment upon distension. (**A**) Scheme showing the potential mechano-adaptation pathway in response to distension in the colonic epithelium, indicating inhibitor and activator targets. (**B**) Scheme of experimental approach for ex vivo culture and distension of colonic explants (**C**) En face ZO-1 images of colonic explants after blebbistatin or vehicle control (DMSO) treatment in ND and D regions. MIP (1- to 3-μm range). Red arrows represent discontinuous ZO-1 staining. Scale bars, 5 μm. (**D**) En face images of colonic explants stained for ZO-1 and pMLC (S19) after Calyculin A or vehicle control (water) treatment in ND and D regions. MIP (1- to 3-μm range). Scale bars, 5 μm. (**E**) Junctional pMLC intensity in Calyculin A- and vehicle control (water)–treated ND and D explants; *n* = 53 to 59 junctions/condition; *N* = 3. (**F**) Fold changes in junctional ZO-1 intensity in distended explants following pretreatment with upstream myosin II inhibitors or Calyculin A. Dotted line indicates the normalized ND average. (**G**) En face images of colonic explants stained for ZO-1 in ND and D after Gd^3+^ or vehicle control (water) treatment. MIP (1- to 3-μm range). Scale bars, 5 μm. (**H**) Junctional pMLC intensity in Gd^3+^ and vehicle control (water)–treated ND and D explants; *n* = 58 to 61 junctions/condition, *N* = 5. (**I**) En face images of colonic explants stained for ZO-1 and pMLC (S19) in ND regions following Yoda1 or vehicle control (DMSO) treatment. MIP (1- to 3-μm range). Scale bars, 5 μm. (**J**) Junctional pMLC intensity in Yoda1-treated and vehicle control (DMSO)–treated ND explants; *n* = 42 to 65 junctions/condition; *N* = 4 (**K**) Fold changes in junctional ZO-1 intensity following pretreatment with mechanosensitive ion channel inhibitors or Piezo1 activator. Dotted line indicates the normalized ND average. Statistical analyses: Mann-Whitney *U* test (J) and Kruskal-Wallis test followed by Dunn’s multiple comparison [(F), (H), and (K)]. BAPTA, 1,2-bis(2-aminophenoxy)ethane-*N*,*N*,*N*′,*N*′-tetraacetic acid.

To target all myosin II isoforms simultaneously, we used blebbistatin, a general myosin II inhibitor known to reduce cortical contractility (*31*). Myosin II inhibition in gut explants completely abolished the recruitment response across all three types of junctions, resulting in aberrant cell and junctional morphology (Fig. 4, C and F, and fig. S6, G to K). To investigate the mechanism underlying the activation of myosin II upon distension, we examined two key pathways: Rho–guanosine triphosphatase–mediated activation of ROCK and calcium-calmodulin-dependent activation of MLCK. Both pathways converge on myosin II by phosphorylating the regulatory light chain (MLC), thereby enhancing contractility. Pharmacological inhibition of either ROCK (Y27632) or MLCK (ML7) blocked MLC phosphorylation and fully suppressed junctional complex recruitment under distension (Fig. 4F and fig. S7, A to P). Conversely, increasing myosin II activity by inhibiting MLC phosphatase with calyculin A led to enhanced phosphorylation of MLC and increased junctional recruitment (ZO-1, 1.5-fold; pMLC, 1.7-fold) compared to mechanical distension alone (Fig. 4, D to F, and fig. S7Q). Collectively, these data suggest that myosin II activation via both ROCK and MLCK pathways is critical for the recruitment of junctional complexes upon distension. Moreover, the fact that weight-mediated distension induces junctional recruitment in colonic organoid monolayers, which lack luminal contents and muscle contractility, indicates that this response is epithelium-autonomous and mechanically triggered (*32*, *33*), rather than driven by chemical cues from feces or muscle contractility.

To investigate whether the observed myosin II recruitment and MLC phosphorylation are mediated via calcium influx upon distension, we inhibited the activity of mechanosensitive ion channels either via gadolinium hexahydrate chloride (Gd^3+^) (*34*) or GsMTx4 (*35*) or blocked calcium entry by chelating extracellular calcium with 1,2-bis(2-aminophenoxy)ethane-*N*,*N*,*N*′,*N*′-tetraacetic acid (BAPTA) (*36*). These treatments abolished both the MLC phosphorylation and the junctional recruitment in response to distension (Fig. 4, G and H and K, and fig. S8, A to H). In agreement, the activation of the mechanosensitive calcium channel Piezo-1 using the chemical agonist Yoda1 (*37*) resulted in myosin II activation and recruitment and the accumulation of junctional ZO-1 (1.72 fold; Fig. 4, I to K, and fig. S8, I and J). Together, our findings indicate that myosin II activation is required for efficient junctional recruitment in response to mechanical distension and support a role for mechanosensitive ion-channel activity and extracellular Ca^2+^ as upstream regulators of this response.

### Junctional reinforcement protects the colonic barrier from mechanical stress-induced breach

During homeostasis, epithelial barrier integrity is maintained by adhesive junctional complexes, primarily TJs, which form a continuous belt-like structure around cells, sealing the paracellular space and preventing luminal translocation. Since junctional recruitment under mechanical stress was impaired when myosin II activity was disrupted, we next asked whether barrier integrity was functionally compromised under these conditions. In both distended NMMIIA-KO tissue and blebbistatin-treated explants, impaired junctional recruitment was accompanied by irregular and discontinuous ZO-1 staining (Figs. 3C and 4C, and fig. S4N), suggesting that failure of myosin II–dependent reinforcement is associated with altered TJ organization. To determine whether these discontinuous junctions represent spatially defined breaches in the epithelial barrier in intact tissue (Fig. 5A), we assessed luminal accessibility of E-cad using a sequential staining assay. Antibodies against the extracellular domain of E-cad were applied to intact, freshly fixed tissue before permeabilization, allowing barrier breaches to be mapped by luminal antibody access to E-cad while preserving the spatial organization of the epithelium (Fig. 5B) (*38*, *39*). Under distension, NMMIIA-KO mice showed a significant increase in junctional accessible E-cad signal compared with control mice (control: 1.06-fold; NMMIIA-KO: 9.40-fold; Fig. 5, C and D), indicating increased junctional breaching under mechanical stress. Similarly, myosin II inhibition increased accessible E-cad signal in distended colonic explants compared with controls (Fig. 5, E to G, and fig. S9, A and B). In addition, the inhibition of mechanosensitive ion channels using gadolinium salts also resulted in barrier breach upon distension (Fig. 5, H to I). These findings indicate that loss of NMMII function—through abolished MLC phosphorylation and/or inhibited calcium influx through mechanosensitive ion channels—compromises barrier integrity under extrinsic mechanical stress. These compromised junctions may allow translocation of luminal contents, including microbial metabolites and toxins, potentially triggering inflammation and gut disorders. Notably, in some distended regions of NMMIIA-KO tissue, we observed junctional fractures accompanied by bacteria in the paracellular space (fig. S8D).

**Fig. 5.**
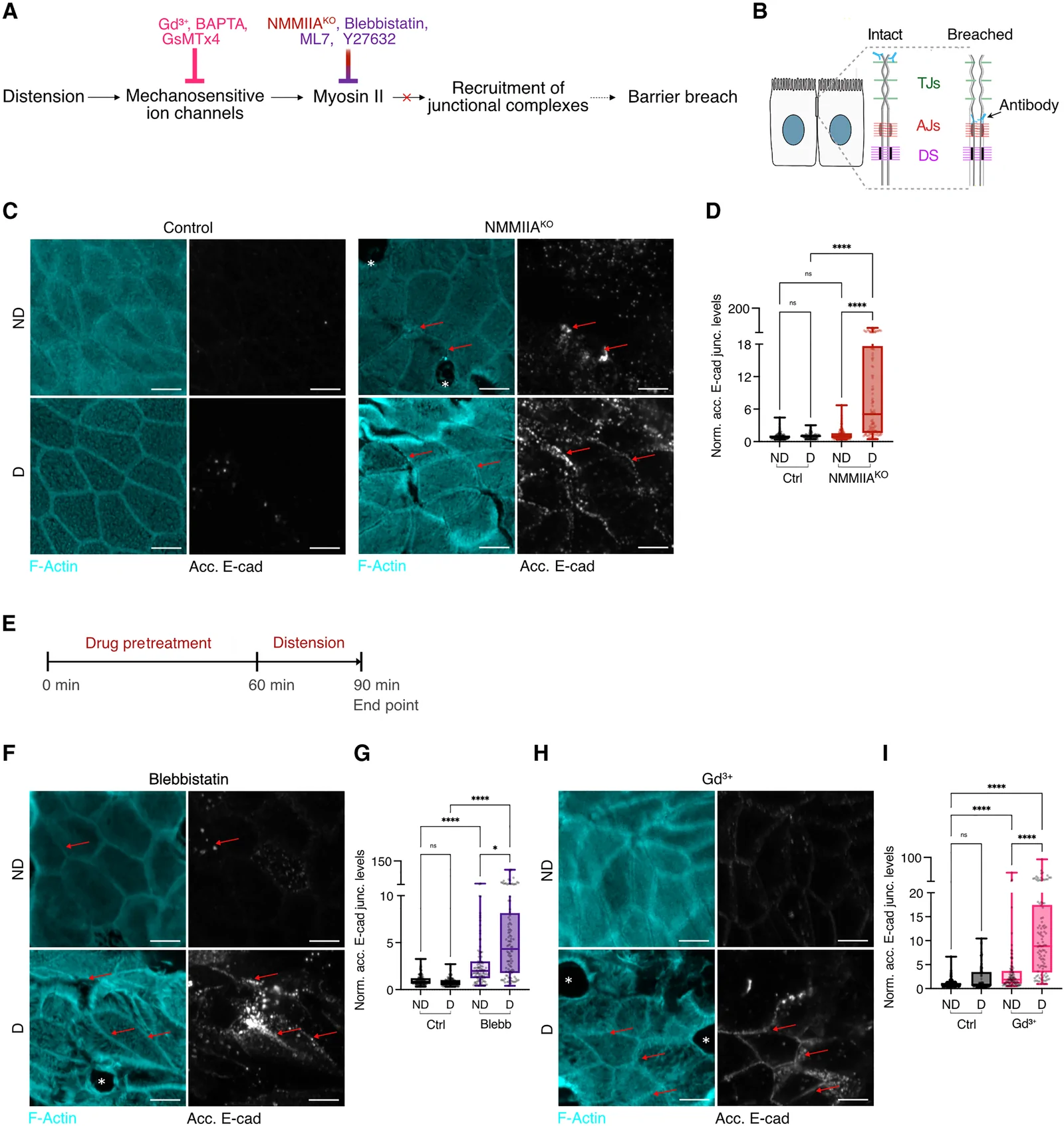
Junctional recruitment protects epithelial barrier integrity during distension. (**A**) Schematic of the proposed mechanoadaptation pathway in colonic epithelium, illustrating how failed junctional recruitment may lead to barrier breach. (**B**) Scheme illustrating the accessible E-cad (acc. E-cad) assay (see Materials and Methods), where E-cad antibody stains only if the TJs are breached. (**C**) En face images of colonic epithelium stained for F-actin and acc. E-cad in control and NMMIIA^KO^ mice from ND and feces-D regions. Red arrows indicate breached junctions. MIP (1- to 3-μm range). Scale bars, 5 μm. (**D**) Junctional acc. E-cad intensity in control and NMMIIA^KO^ mice from ND and feces-D regions; *n* = 32 to 68 junctions/condition, *N* = 3. (**E**) Scheme showing experimental approach for accessible E-cad assay in ex vivo culture. (**F**) En face images of colonic explants stained for acc. E-cad in ND and D regions following blebbistatin or vehicle control (DMSO) treatment. MIP (1- to 3-μm range). Scale bars, 5 μm. (**G**) Junctional acc. E-cad intensity in blebbistatin- and vehicle control (DMSO)–treated ND and D explants; *n* = 34 to 69 junctions/condition; *N* = 3. (**H**) En face images of colonic explants stained for acc. E-cad in ND and D regions following Gd^3+^ or vehicle control (water) treatment. MIP (1- to 3-μm range). Scale bars, 5 μm. (**I**) Junctional acc. E-cad intensity in Gd^3+^ and vehicle control (water)–treated ND and D explants; *n* = 34 junctions/condition; *N* = 3. Statistical analyses: Kruskal-Wallis test followed by Dunn’s multiple comparison [(D), (G), and (I)]. *: goblet cell.

In conclusion, we show that colonic mucosa undergoes coordinated cell- and tissue-scale remodeling to accommodate feces, with junctional complexes responding by recruiting their constituent proteins to reinforce the barrier. Our findings highlight NMMII as a key mediator of this recruitment, activated by extracellular calcium influx through mechanosensitive ion channels. Loss of junctional recruitment response leads to barrier defects under mechanical stress, underscoring the critical role of junctional reinforcement in maintaining gut epithelial integrity.

## DISCUSSION

Few studies have investigated how different adhesive junctions behave and interact under mechanical stress, and these have mostly relied on loss-of-function approaches in epithelial model systems (*32*, *40*) or embryonic tissues (*41*). For example, perturbation of AJ-associated mechanotransduction can impair TJ organization and barrier function (*42*, *43*), while desmosomal disruption can alter RhoA-dependent responses to mechanical tension (*32*), illustrating reciprocal mechanical coupling between junctional systems. TJs and AJs are also molecularly linked through scaffolding proteins such as ZO-1, which can couple barrier assembly to α-catenin–dependent cell-cell adhesion and regulate tension at cadherin-based junctions (*44*–*46*). Here, we report that during physiological feces-mediated colon distension in adult mice, epithelial cells experience mechanical forces leading to recruitment of proteins from all three types of junctional complexes (TJs, AJs, and desmosomes). We have analyzed the coordination and temporal regulation of junctional recruitment across all three junction types in the adult colon in vivo. This revealed protein-specific temporal profiles of junctional enrichment in response to extrinsic mechanical force. ZO-1, E-cad, DSC2, and PG were recruited within 5 min of distension and remained elevated for up to 30 min, suggesting a sustained mechanoadaptive response. Given the role of ZO-1 as a core TJ scaffolding protein, its rapid and sustained recruitment is expected to contribute to preserving barrier integrity under mechanical stress. PG—which can bind to both AJs and desmosomes—has previously been identified as a mechanosensitive protein in Xenopus mesendoderm cells, where it plays a key role in the reorganization of keratin intermediate filaments toward sites of mechanical stress (*41*). Therefore, the sustained levels of PG during distension in the gut epithelium may support both AJ and desmosome assembly, potentially helping to couple cadherin-based adhesion to desmosome-keratin reinforcement.

In contrast, DP and K8 showed progressive accumulation, peaking around 15 min of distension, with higher fold changes than those observed for TJ and AJ proteins. This observation is consistent with a recent in vitro study demonstrating that keratin intermediate filaments mediate epithelial strain-stiffening under large mechanical deformation, providing structural reinforcement against sustained mechanical force (*47*). Together, these temporally distinct responses suggest that junctional reinforcement is coordinated across adhesive systems but does not occur through uniform recruitment of all components. This temporal separation is reminiscent of calcium-switch models of desmosome assembly, in which the recruitment of desmosomal components to cell-cell contacts and the subsequent maturation of DP-intermediate filament coupling can occur through partially distinct steps. The sustained enrichment of PG may fit within this framework, as PG can associate with both AJs and desmosomes (*41*, *48*) and could contribute to maintaining adhesive coupling while DP and K8 undergo more dynamic remodeling. Its position at the interface between junctional systems is therefore consistent with a role for PG as a mediator of junctional cross-talk in response to mechanical force (*49*, *50*).

We further showed that NMMII activity governs mechanoadaptation of all adhesive junctions in the gut epithelium, including desmosomes and their associated intermediate filaments. This positions NMMII as an early regulatory hub of epithelial adhesion under mechanical stress, potentially coordinating cross-talk between TJs, AJs, and desmosomes. Part of this coordination may occur through the shared perijunctional actomyosin network, which links TJ and AJ organization. Our α-catenin and pMLC data further support this idea by showing that distension is associated with force-dependent AJ loading and actomyosin activation. However, our data also suggest that this mechanically integrated network extends to desmosomes, possibly through cytolinkers such as dystonin and plectin, which have been implicated in connecting actin filaments to desmosomes and maintaining junctional tension (*32*, *51*). Together with recent work showing that desmosome-intermediate filament coupling can feed back onto AJ mechanotransduction/RhoA signaling and that cortical tension regulates desmosomal protein turnover and composition (*32*, *52*), our findings suggest that NMMII activity may regulate desmosomal reinforcement by modulating the organization, turnover, or trafficking of plaque and cadherin components, including DP and desmosomal cadherins. Future work will be required to determine whether these mechanisms mediate the rapid and reversible desmosomal adaptation observed in the distended gut epithelium.

We observed accumulation and activation of NMMII in the perijunctional space during colonic distension; similar phenomena have been described at junctions during *Drosophila* development, where the stimulation of contractility promotes myosin recruitment and actomyosin remodeling (*53*, *54*). The mammalian gut epithelium expresses two relevant NMMII isoforms: NMMIIA, predominantly associated with AJs and exhibiting a low duty ratio that enables dynamic contractile force generation, and NMMIIC, which localizes to the terminal web and regulates microvillar architecture (*27*, *28*, *55*). Upon distension, NMMIIA gradually accumulated at junctions, peaking at 15 min. This kinetic profile coincided with maximal transient apical constriction and was paralleled by increased pMLC S19 enrichment, indicating the activation of the apical/perijunctional actomyosin network. The close temporal association between NMMIIA accumulation, pMLC enrichment, and apical area reduction suggests that NMMIIA-dependent contractility contributes to the epithelial shape changes induced by distension, potentially through both junctional and apico-medial actomyosin pools. Notably, the 15-min peak also coincided with maximal recruitment of the more “progressive” responders, including DP and K8, suggesting that NMMIIA-mediated contractility may drive, or at least facilitate, their junctional accumulation during mechanical loading. NMMIIC also relocalised to junctions in response to force. Since NMMIIC is normally enriched at the terminal web, its junctional accumulation may reflect broader apical actomyosin remodeling and coupling between the apical cortical cytoskeleton and junctional complexes under mechanical load. In NMMIIA-KO tissue, NMMIIC was already detectable at junctions and increased further upon distension, together with residual pMLC enrichment. Although this does not demonstrate functional compensation, it suggests that NMMIIC may contribute to a remaining actomyosin response when NMMIIA is lost. Consistent with this possibility, the broad pharmacological inhibition of myosin II activity in colon explants abolished junctional recruitment. Thus, NMMIIA appears to be the major isoform required for efficient junctional reinforcement, while NMMIIC may provide a partial or secondary contribution to residual actomyosin activity and junctional adaptation under mechanical load. The inhibition of upstream myosin pathways—either via the Rho-ROCK pathway or MLCK—was sufficient to abolish recruitment across all three types of adhesive junctions upon force, while increasing myosin activity (using calyculin A) enhanced recruitment of junctional proteins, even in the absence of applied force. Our results suggest that these pathways converge on NMMIIA-mediated mechanotransduction rather than acting as fully redundant inputs.

We showed that NMMIIA-mediated junctional reinforcement requires extracellular Ca^2+^ availability and mechanosensitive ion-channel activity, consistent with calcium signaling promoting myosin contractility through activation of RhoA signaling and the calmodulin/MLCK pathway (*7*, *55*–*57*). Calcium signaling in epithelial cells is a key mediator of mechanotransduction, converting mechanical forces into biochemical signals via mechanosensitive ion channels. Recent studies have shown that Piezo1 can associate with AJs or adhesion-associated cortical complexes in epithelial cells, suggesting that force-bearing junctions may serve as privileged sites for mechanosensitive Ca^2+^ signaling (*58*, *59*). This positioning could favor rapid, spatially restricted Ca^2+^ microdomains near the junctional actomyosin belt, thereby promoting local MLCK/NMMII activation and coordinated reinforcement of TJ, AJ, and desmosomal components. In this context, determining whether Piezo1 is similarly organized at junctions in the gut epithelium, and whether this spatial organization tunes the speed or strength of junctional reinforcement, will be an important direction for future work. A recent study of epithelial Piezo loss in the gut in vivo reported severe defects in stem cell differentiation, resulting in diarrhea, weight loss, and early lethality (*60*). However, the more immediate role of Piezo1-dependent mechanosensitive signaling in acute junctional dynamics and barrier integrity remained unclear.

Our pharmacological experiments suggest that mechanosensitive calcium influx acts upstream of actomyosin activation and junctional reinforcement during colonic distension. In particular, Piezo1 activation was sufficient to increase MLC phosphorylation and promote ZO-1 junctional recruitment in nondistended explants, whereas inhibition of stretch-activated channels attenuated these responses under distension. Together, these findings suggest that mechanical distension engages mechanosensitive ion-channel–dependent Ca^2+^ signaling and actomyosin regulation to promote junctional reinforcement. However, this response is likely to integrate additional force-sensitive mechanisms, including α-catenin conformational changes and Rho-ROCK signaling, which may contribute to junctional reinforcement independently of global intracellular Ca^2+^ changes.

This reinforced junctional state was maintained only under sustained extrinsic mechanical load: Upon its removal, junctional protein levels rapidly reverted to baseline levels, indicating that reinforcement is dynamically maintained rather than permanently stabilized. This dynamic regulation may arise from tension-induced conformational changes in junctional components (*9*, *61*, *62*) or from force-mediated, phosphorylation-dependent mechanisms, as the phosphorylation states of PG and DP are known to regulate desmosome assembly and disassembly and intermediate filament coupling (*12*, *18*, *19*, *63*). In line with this, we observed increased junctional phosphorylation of DP at Ser^2849^ upon distension, suggesting that desmosome reinforcement is accompanied by active remodeling of the DP-keratin linkage. This may help explain the dynamic behavior of DP and K8 during distension, including their partial attenuation at later time points and rapid decrease after force removal. The rapid reversion of junctional levels to baseline after force removal in the gut epithelium may limit the energetic cost of maintaining a reinforced junctional state, since this response relies on adenosine 5′-triphosphate–dependent processes such as actin polymerization, actomyosin contractility, protein trafficking, and signaling (*64*–*66*).

Last, our findings establish junctional recruitment as a mechano-adaptive response to mechanical stress, essential for preserving barrier integrity under mechanical challenge. This mechanism may be particularly relevant in gut pathologies such as toxic megacolon, severe constipation, luminal obstruction by tumors, and fibrotic strictures in inflammatory bowel disease, where excessive mechanical distension could worsen disease outcomes (*67*–*69*). Consistent with a broader protective role for epithelial NMMIIA, the chronic loss of NMMIIA in a gut epithelium–specific constitutive KO model was reported to increase intestinal permeability under basal conditions and to further exacerbate barrier defects during experimental colitis (*70*). In our study, the luminally accessible E-cad assay provided evidence of epithelial barrier breaches in vivo when NMMIIA-dependent junctional reinforcement was impaired, while primary colonic epithelial monolayers recapitulated the mechanically induced junctional response, supporting its epithelial-intrinsic nature. A limitation of the present study is that we did not directly measure barrier function in these primary epithelial monolayers. Thus, whether this mechano-adaptive junctional reinforcement is sufficient to preserve transepithelial barrier function in isolated primary colonic epithelium remains to be tested directly.

In conclusion, we demonstrate that the adult colonic epithelium mounts a robust mechano-adaptive response to mechanical stress, whereby NMMII-dependent actomyosin activity, supported by mechanosensitive calcium influx, coordinates junctional reinforcement to preserve barrier integrity. This reveals how the gut epithelium actively maintains barrier function under physiological mechanical load.

## MATERIALS AND METHODS

### Mice

All mouse husbandry and handling procedures were approved and conducted in accordance with European and National Regulations (protocol authorization APAFIS #38994-2023062718345638, APAFIS #14513-2018040313435341 v6.). Mice were housed in the Specific and Opportunistic Pathogen-Free (SOPF) facility at the CBI and CREFRE SOPF facility of INSERM Purpan. Experiments were performed on age-matched littermates (8 to 12 weeks old) of both sexes, as no phenotypic differences were observed between males and females. C57BL/6N mice were obtained from Charles River Laboratories (France). Villin: Cre^ERT2^ mice were crossed with Myh9^flox^ (Mutant Mouse Resource and Research Center, stock 032096-UNC) to generate NMIIA^KO^ mice. Villin: Cre^ERT2^ and NMIIA^GFP^ mice were provided by D. Matić Vignjević (Institut Curie).

### Tamoxifen treatment

To induce intestinal epithelium-specific KO of Myh9 (NMIIA), Villin: Cre^ERT2^/Myh9^fl/fl^, and Myh9^fl/fl^^ control mice were injected with 100 μl of tamoxifen (50 mg/kg; Euromedex, SE-S1238) intraperitoneally once daily for five consecutive days and culled 3 days after the last injection.

### Antibodies

For immunofluorescence, we used the following antibodies: rat anti-ZO-1 (Millipore, MABT11) at 1:200, rat anti-E-cad (Invitrogen, 1319800) at 1:200, rabbit anti-occludin (Proteintech, 27260-1-AP) at 1:50, mouse anti-α-catenin (Santa Cruz Biotechnology, SC-9988) at 1:50, rat anti–α-18 (a kind gift from Akira Nagafuchi) at 1:100, mouse anti–β-catenin (Santa Cruz Biotechnology, SC-7963) at 1:50, rabbit anti-NMMIIC (Proteintech, 909802) at 1:100, rabbit anti-pMLC (Ser19) (Cell Signaling Technology, 3671) at 1:100, sheep anti-DSC2 (Biotechne, AF7490) at 1:50), mouse anti–γ-catenin (PG) (Santa Cruz Biotechnology, SC-514115) at 1:50, mouse anti-DP1/2 (Progen, 651109) at 1:50, rabbit anti-DP pS2849 (Rockland, 600-401-J65) at 1:100, rabbit anti-cytokeratin 8 (Abcam, ab240986) at 1:200, rabbit anti-laminin (Merck, L9393) at 1:200, and UEA-1 fluorescein isothiocyanate (Invitrogen, L32476) at 1:100. All the secondary antibodies (Molecular Probes) were used at a 1:200 dilution. F-actin was labeled with Rhodamine- or Alexa Fluor 647–conjugated phalloidin (Invitrogen, R415 and A30107) at 1:200. DNA was stained using 4′,6-diamidino-2-phenylindole (DAPI) (Sigma-Adrich, D9452) at 5 μg/ml. For Western blotting, the same antibodies as those listed for immunofluorescence were used. Additional antibodies specific to Western blotting include: rabbit anti-ZO1 (Proteintech, 21773-1-AP) at 1:10,000, rabbit anti-NMMIIC (Cell Signaling Technology, 8189 T) at 1:5000, rabbit anti-NMMIIA (Biolegend, 909802) at 1:5000, rabbit anti–cytokeratin 8 (Abcam, ab227644) at 1:1000, rabbit anti-GAPDH (Sigma, G9545) at 1:40000. Horseradish peroxidase (HRP)–conjugated secondary antibodies against rabbit, mouse, and rat IgG (1:5000) were purchased from Jackson Immunoresearch.

### Tissue fixation and sectioning

Mice were culled as indicated, and the entire colon were isolated and fixed immediately in 4% paraformaldehyde (Electron Microscopy Sciences) in Phosphate-buffered saline (PBS) for 1 hour at room temperature (RT), without flushing the luminal contents. After fixation, tissues were rinsed three times with 1× PBS and then processed by sectioning. For transverse sections, the fixed colon was cut into 150- to 200-μm-thick slices using a vibratome (Leica; VT1200). For en face sections, the colon was cut open longitudinally along its length, and small rectangular sections (≈4 mm^2^) were dissected and isolated (fig. S1A).

### 3D immunofluorescence

The fixed tissue sections were permeabilized with 1% Triton X-100 (Sigma-Aldrich) in PBS for 1 hour at RT followed by incubation with primary antibodies in washing buffer (0.2% Triton X-100/PBS) overnight at RT under gentle agitation. After incubation, the samples were washed three times for 1 hour each with the washing buffer and then incubated with secondary antibody, DAPI and with or without phalloidin, in the washing buffer. Following three washes of 1 hour each, the sections were mounted on slides so that the epithelial surface/plateaus were positioned directly beneath the coverslip using mounting medium (Aqua-Poly/Mount, Polysciences), according to the manufacturer’s instructions. This ensured comparable imaging depths across all samples, thereby minimizing variability in fluorescence intensity measurements arising from differences in optical path length.

### Transmission electron microscopy

The colon was isolated as described above and fixed without flushing the luminal contents using a final concentration of 2.5% glutaraldehyde and 2% paraformaldehyde in 0.1 M cacodylate buffer (pH 7.2, EMS) for 30 min at room temperature, followed by an overnight incubation at 4°C. Postfixation was performed for 1 hour at room temperature with 1% osmium tetroxide (OsO_4_) and 1.5% potassium ferricyanide [K_3_Fe(CN)_6_] in 0.1 M cacodylate buffer, followed by treatment with 2% aqueous uranyl acetate for 1 hour at room temperature. Samples were then dehydrated through a graded acetone series and embedded in Epon resin (EMS, Embed 812). After 48 hours of polymerization at 60°C, ultrathin sections (80-nm thick) were mounted onto single-slot Formvar-coated grids and stained with Uranyless (EM-grade) and lead citrate (EM-grade). Grids were examined using a JEOL JEM-1400 transmission electron microscope (JEOL Inc., Peabody, MA, USA) operated at 80 kV, and images were acquired with a Gatan Rio 9 digital camera (Gatan Inc., Pleasanton, CA, USA)

### Colon relaxation assay

Colon was isolated as described earlier, and segmented into regions with feces (distended) or without feces (nondistended). Fecal pellets were either gently removed from distended fragments using forceps or left inside, after which all samples including nondistended, distended, and relaxed fragments (fragments after fecal removal) were incubated in Leibovitz’s L-15 medium (Gibco, 11415064) at 37°C for 5 min, followed by fixation and processing as described in the 3D immunofluorescence staining protocol.

### Catheter-mediated in vivo distension

Colonic distension was performed as previously described (*23*). Briefly, the mice were anaesthetized via i.p. injection of ketamine and xylazine (6.7 mg/Kg and 133 mg/Kg, respectively). A Fogarty Thru-Lumen Embolectomy Catheter (4F; Edwards Lifesciences) with a deflated balloon was inserted 3 cm proximally from the anus. The balloon was then inflated with 1 ml water to a pressure of 80 mmHg/10.6 kPa, which was maintained for either 5, 15, or 30 min. After each time point, mice were culled, and the colon containing the inflated balloon was carefully isolated and fixed in 4% paraformaldehyde for 1 hour at RT. Following fixation, the tissue was processed for 3D immunofluorescence as described above.

### Explant culture and treatments

Colonic explant cultures were performed with slight modification to previously described method (*29*). Briefly, mice were euthanized, and the nondistended part of the colon was isolated and opened up longitudinally. Small sheets of tissue (≈4 mm^2^) were cut and placed on a hydrophilic PTFE membrane insert with pore size of 0.4 μm (Millipore, PICM0RG50), epithelium facing the membrane. The explant medium consists of DMEM-F12 (Gibco, 12634010) supplemented with 2.5% fetal bovine serum (FBS), 1X GlutaMAX (Thermo Fisher Scientific, 11574466), 1X insulin-transferrin-selenium solution (Gibco, 41400045), transferrin (100 μg/ml; Santa Cruz Biotechnology, 391098), murine EGF (10 ng/ml; PeproTech, 315-09), and 1% anti-anti (Gibco, 15240062). Explants were cultured at 37°C in a humidified atmosphere of 95% O_2_ and 5% CO_2_, in the presence or absence of pharmacological inhibitors/chemical compounds.

To apply mechanical stimulation, tissues were either left nondistended or subjected to mechanical loading by placing a 2-g sterile, static weight directly on top of the explant’s serosal side for 30 min (≈5 kPa for a 4 mm^2^ surface area), adapting approaches previously used in epithelial mechanics (*71*–*73*). The estimated stress assumes uniform force distribution over the explant surface and does not correct for local deformation or tissue stiffness. Tissues were then fixed and processed for staining as described above.

For the combined pharmacological and mechanical treatments, the explants were pretreated for 1 hour with one of the following inhibitors or compounds: 170 μM blebbistatin (Selleck Chemicals, S7099), 100 μM Y27632 (Y27) (Santa Cruz Biotechnology, sc-281642A), 100 μM ML-7 (Sigma-Aldrich, 475880), 100 μM calyculin-A (Abcam, ab141784), 30 μM GsMTx4 (MedChemExpress, HY-P1410), or 100 μM gadolinium (III) chloride (Gd^3+^) (Sigma-Aldrich, G7532). Following pretreatment, tissues were maintained under either nondistended or distended conditions for 30 min. For treatment with 500 μM Yoda1 (Sigma-Aldrich, SML1558), explants were incubated for 15 min in the nondistended condition. For 2 mM BAPTA (Millipore, 196418) treatment, the compound was added directly to the media, and samples were immediately subjected to distension or maintained nondistended for 30 min.

### Accessible E-cad assay

The accessible E-cad assay was performed as previously described (*38*, *39*). Briefly, fixed tissue sections were incubated with a primary antibody against the extracellular domain of E-cad (ECCD-2) in PBS at RT under gentle agitation overnight. After three 1-hour washes in PBS, the samples were incubated with a secondary antibody in PBS overnight at RT, followed by a further three 1-hour washes. The tissues were then permeabilised with 1% Triton X-100 in PBS for one hour at RT and subsequently processed using the 3D immunofluorescence staining protocol described earlier.

### Organoid culture

#### Intestinal crypt isolation and 3D organoid culture

Wild-type mice (2 months old) were euthanized, and the colon was isolated, cut longitudinally, washed in PBS, and cut into two to three pieces. Tissue was dissociated in PBS supplemented with 15 mM EDTA and 1 M DTT for 20 min at 4°C, followed by incubation in PBS-EDTA (15 mM) for 15 min at 37°C. The sample was then transferred to DMEM/F12 (Gibco, 12634010) and shaken vigorously to release crypts into suspension. After centrifugation at 1200 rpm at 4°C for 5 min, the pellet was resuspended in 5 ml of PBS supplemented with 2% antibiotic-antimycotic solution (Gibco, 15240062) and passed through a 70-μm cell strainer (Corning). The suspension was centrifuged again at 1200 rpm at 4°C for 5 min. The pellet was resuspended in a 1:1 mixture of growth factor–reduced Matrigel (Corning, 356231) and WRN medium and plated as 40-μl drops in 24-well plates. After polymerization for 25 min at 37°C in a humidified atmosphere with 5% CO_2_, wells were supplemented with 350 μl of WRN medium. Organoids were passaged every 3 to 4 days by mechanical dissociation.

The WRN organoid medium consisted of 50% Dulbecco’s modified Eagle’s medium/F12 and 50% L-WRN conditioned medium (*74*), supplemented with 1× GlutaMAX (Thermo Fisher Scientific, 11574466), 2% antibiotic-antimycotic solution, murine epidermal growth factor (50 ng/ml; PeproTech, 315-09), and 1× B27 supplement (Gibco, 11500446).

#### 2D organoid culture, mechanical distension, and 3D immunofluorescence

Before generation of 2D organoids, 3D organoids were pretreated to promote proliferation and multipotency by culture in WRN medium supplemented with 3 μM CHIR 99021 (PeproTech, 2520691) and 1 μM valproic acid (Sigma-Aldrich, P4543) for at least 3 days.

2D organoid culture was performed as previously described (*30*). Briefly, 3D organoids were mechanically dissociated and the resulting cells were seeded onto Transwell inserts (Costar, 3413) with 0.4 μm polycarbonate membranes coated with 10% Matrigel in PBS for 20 min at 37°C. Cells were cultured in WRN medium supplemented with 10 μM Y-27632 (Santa Cruz Biotechnology, CAS 129830-38-2) for 7 days at a liquid-liquid interface (LLI), followed by 5 days at an air-liquid interface (ALI). At day 5, mechanical stimulation was applied to the monolayers by placing a 1-g sterile static weight directly onto the cells for 30 min. Cells were maintained either under nondistended or distended (mechanically loaded) conditions.

For immunofluorescence, samples were fixed in 4% paraformaldehyde (Electron Microscopy Sciences) for 10 min at room temperature and washed three times with PBS. Samples were then permeabilized with 0.1% Triton X-100 for 10 min, followed by blocking in 10% FBS for 1 hour at RT. Primary antibodies, diluted in 10% FBS / 0.1% Triton X-100, were added and incubated overnight at 4°C. After three washes of 5 min each in 0.1% Triton X-100, secondary antibodies and phalloidin, diluted in 10% FBS/0.1% Triton X-100, were added and incubated for 1 hour at RT. Samples were washed three times for 5 min each and mounted onto glass slides using Aqua-Poly/Mount mounting medium (Polysciences).

### Western blotting

The mouse colons were isolated and opened longitudinally. The distended regions containing feces were either separated from the nondistended regions, or the entire colon was washed in PBS and cut into multiple fragments. The tissues were incubated in a solution of 30 mM EDTA and 1 mM dithiothreitol in PBS for 20 min on ice, followed by 30 mM EDTA/PBS for 15 min at 37°C. Samples were then vigorously agitated and centrifuged at 1200 rpm for 5 min at 4°C to isolate epithelial cells. Cells were lysed in radioimmunoprecipitation assay buffer containing 50 mM tris base, 150 mM NaCl, 1% SDS, 1% sodium deoxycholate, 1% Triton X-100, protease inhibitors (Roche, 11836170001), and phosphatase inhibitors I and II (Bimake, B15001 and B15002). Samples were sonicated for 20 s and centrifuged at 12,000*g* for 10 min at 4°C. Protein concentration was measured using the DC assay (Bio-Rad, 5000112), and 25 μg of protein was mixed with 4× Laemmli buffer, boiled at 95°C for 5 min, and loaded onto 4 to 20% SDS–polyacrylamide gels (Bio-Rad, 4568095) under reducing conditions. Proteins were transferred to nitrocellulose membranes (Bio-Rad, 1704271), blocked with blocking buffer (Bio-Rad, 12010020) for 30 min, and incubated with primary antibodies for 1 hour at room temperature or overnight at 4°C. After washing, membranes were incubated with HRP-conjugated secondary antibodies for 1 hour at room temperature. Protein bands were revealed by ECL (chemiluminescence, Bio-Rad) and imaged with a ChemiDoc imaging system (Bio-Rad).

### Confocal 3D imaging

All images were acquired from the plateau regions of the distal colon using transverse sections to visualize the apicobasal axis of epithelial cells, and sheet-like sections and explants to image the epithelium from the apical (luminal) side. Imaging was performed using a LSM 880 (Zeiss) inverted laser scanning microscope equipped with 405-, 488-, 561-, and 633-nm lasers. Objectives used included 20× (*z*-step size: 0.8 μm), 40× (*z*-step size: 0.4 μm) oil immersion, and 63× (*z*-step size: 0.3 μm) oil immersion, with images captured at a resolution of 1024 by 1024 pixels.

### High-resolution (Airyscan) 3D tissue imaging

Images were acquired with LSM 880 (Zeiss) inverted laser scanning microscope equipped with Airyscan module. A 63× (*z*-step size: 0.2 μm) oil immersion objective was used, and images were captured at a resolution of 1024 by 1024 resolution. Images were further processed using the Airyscan processing module in ZEN software.

### Image analysis

All images were processed and analyzed using ImageJ software (NIH). Optical z-steps (0.3-μm intervals) were acquired to account for differences in cell height and tissue folding. Maximum intensity projections of image stacks (1- to 3-μm range) from en face sections, encompassing apical junctional complexes, were used for quantification. The *z*-stack range for TJ and AJ proteins in each region of interest was defined to encompass the perijunctional F-actin signal, ensuring that the F-actin belts of neighboring cells were distinguishable. Similarly, for desmosomes and associated proteins, the Z-stack range was defined to include the most-apical desmosome DP staining. Junctional fluorescence intensity was manually quantified using the line scan function by drawing a segmented line (10 pixels wide) orthogonal to randomly selected homotypic junctions, extending from vertex to vertex of the cell. To correct for cytoplasmic background, an equivalent line was drawn in the adjacent cytoplasm, and its intensity was subtracted from the junctional signal. For perijunctional proteins such as NMMIIA, NMMIIC, and K8, intensity measurements were performed by line scans (10 pixels wide) encompassing the perijunctional regions between vertices. All intensity values were normalized to their respective nondistended controls within each experiment to calculate fold changes. The quantitative analysis of junctional and cytoplasmic protein distribution within cell was assessed using the Plot profile tool in ImageJ. A line of constant length was drawn perpendicular to the junctions, extending into the cytoplasm, and the average intensity was plotted along the normalized length.

Apical cell area was measured by segmentation on ZO-1 staining using Tissue Analyzer plugin in ImageJ and represented as area heatmap using Imaris software (Imaris 10.2, Oxford instruments).

Transverse images of colonic tissue were used to quantify cell and intercryptal aspect ratios. Cell aspect ratio was defined as the ratio of cell height to width in plateau regions, with F-actin staining used to demarcate cell boundaries and laminin to mark the basement membrane. Cell height was measured from the basement membrane to the tip of the microvilli, and width was measured as the distance between opposing lateral membranes at the cell midpoint. The intercryptal aspect ratio was calculated as the ratio of intercryptal region height to plateau width, with plateau width defined as the distance along the epithelial surface between adjacent crypt openings, and intercryptal height measured from the base of the intercryptal region to the epithelial surface.

### Statistics

Statistical analysis was performed using GraphPad Prism 10.4.2. All experiments were independently repeated at least three times unless stated otherwise, and *N* represents the number of independent experiments. Normality of data was assessed using the Shapiro-Wilk test. For comparisons between two groups, normally distributed data were analyzed using unpaired Student’s *t* tests, while non-normally distributed data were analyzed using the Mann-Whitney test. For comparisons involving three or more groups, one-way ANOVA followed by Bonferroni’s post hoc test was applied to normally distributed samples. For non-normally distributed samples, the Kruskal-Wallis test followed by Dunn’s multiple comparison test was used. Data are presented as box-and-whisker plots—the box represents the interquartile range (25th to 75th percentiles), the central line indicates the median, and the whiskers denote the minimum and maximum values. Bar charts represent average values from individual experiments and error bars indicate SEM. Statistical parameters—including sample size (*n*), independent experiments (*N*), statistical test performed, and significance values—are provided in the corresponding figure legends. Symbols used are: not significant (ns): *P* > 0.05; *P ≤ 0.05; **P ≤ 0.01; ***P ≤ 0.001; and ****P ≤ 0.0001.
