# Supplementary material for: Robust pan-junctional reinforcement preserves the gut epithelial barrier under mechanical stress
Source: Sci Adv. 2026 Oct 30;12(44):eaed3122. doi: 10.1126/sciadv.aed3122 (PMC13722002; doi:10.1126/sciadv.aed3122)
Supplement: Supplementary file 1 [file sciadv.aed3122_sm.pdf]

Science Advances
AAAS

Supplementary Materials for

Robust pan-junctional reinforcement preserves the gut epithelial barrier under mechanical stress

Vishnu Krishnakumar *et al.*

Corresponding author: Denis Krndija, denis.krndija@utoulouse.fr

Sci. Adv. **12**, eaed3122 (2026)
DOI: 10.1126/sciadv.aed3122

This PDF file includes:

Figs. S1 to S9

Supplementary Figures

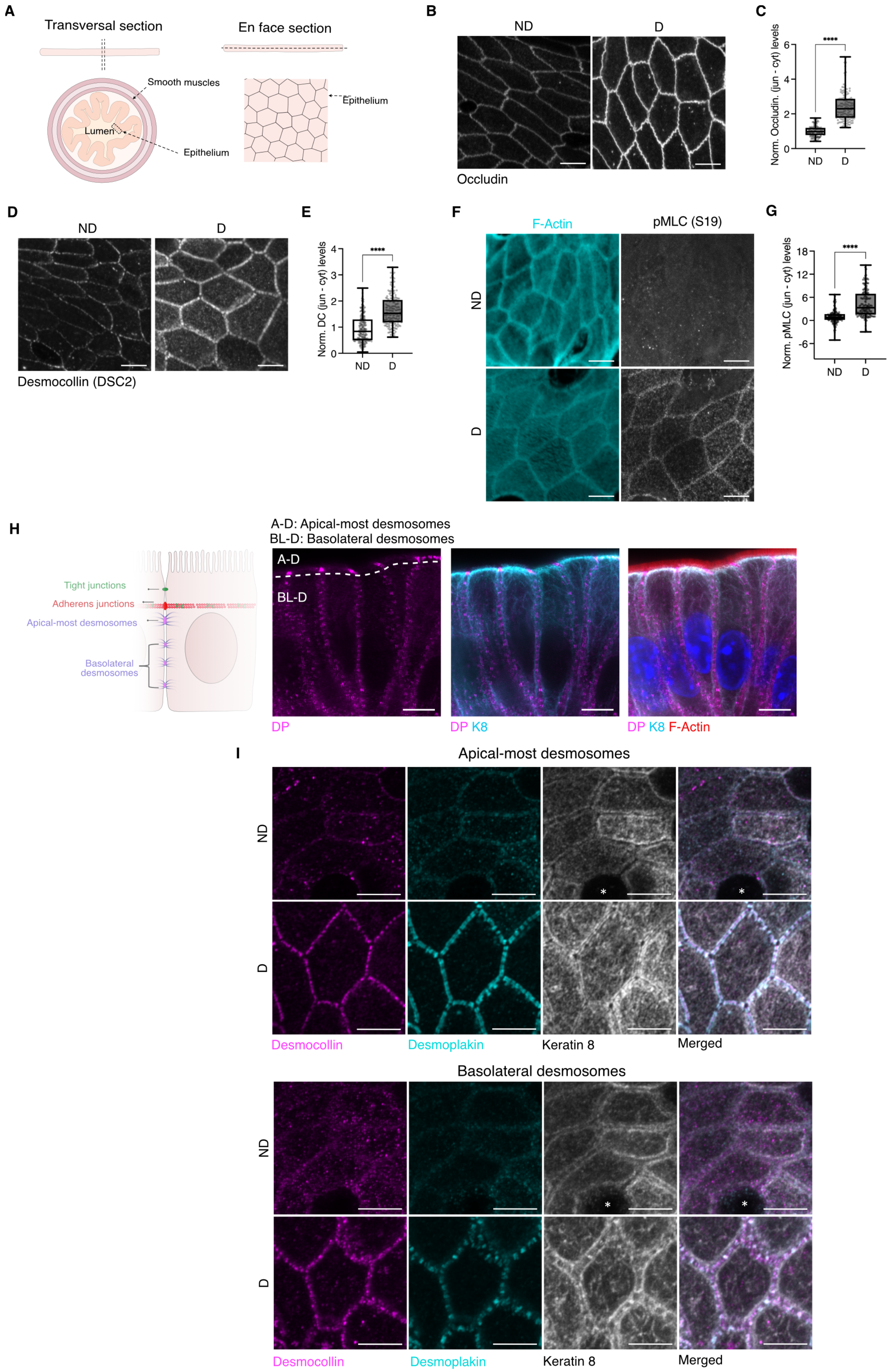
A
Transversal section
En face section
Smooth muscles
Lumen
Epithelium
Epithelium
B
ND
D
Occludin
C
Norm. Occludin. (jun - cyt) levels
****
ND
D
D
ND
D
Desmocollin (DSC2)
E
Norm. DC (jun - cyt) levels
****
ND
D
F
F-Actin
pMLC (S19)
ND
D
G
Norm. pMLC (jun - cyt) levels
****
ND
D
H
Tight junctions
Adherens junctions
Apical-most desmosomes
Basolateral desmosomes
A-D: Apical-most desmosomes
BL-D: Basolateral desmosomes
A-D
BL-D
DP
DP K8
DP K8 F-Actin
I
Apical-most desmosomes
ND
D
Desmocollin
Desmoplakin
Keratin 8
Merged
Basolateral desmosomes
ND
D
Desmocollin
Desmoplakin
Keratin 8
Merged

Fig. S1. **(A)** Schematic representation of transverse and *en face* sectioning of the mouse colon. **(B)** Representative *en face* images of colonic epithelium, stained for occludin in ND and D regions. Maximum Z-projections, 1-3 μm. Scale bar: 5 μm. **(C)** Box plots showing junctional intensity for occludin (n=48-51 junctions/condition) in ND and D regions, N=3 experiments **(D)** Representative *en face* images of colonic epithelium, stained for desmocollin in ND and D regions. Maximum Z-projections, 1-3 μm. Scale bar: 5 μm. **(E)** Box plots showing junctional intensity for desmocollin (n=53-73 junctions/condition) in ND and D regions, N=3 experiments. **(F)** Representative *en face* images of colonic epithelium, stained for F-actin (cyan) and pMLC S19 (grey) in ND and D regions. Maximum Z-projections, 1-3 μm. Scale bar: 5 μm. **(G)** Box plots showing junctional intensity for pMLC(S19) (n=42-60 junctions/condition) in ND and D regions, N=4 experiments. **(H)** Left: Schematic representation of the main adhesive junctional complexes: tight junctions (TJs), adherens junctions (AJs) and desmosomes (Ds). Right: Representative transverse image of distended colonic epithelium stained for DP (magenta), K8 (cyan) and F-actin (red). Apical-most desmosomes are located immediately below the F-actin/AJ level, demarcated by the white dashed line, whereas basolateral desmosomes are located below. **(I)** Representative *en face* images of colonic epithelium stained for DC (magenta), DP (cyan) and K8 (grey) showing apical desmosomes (top); maximum Z-projection (1-3 μm range) and basolateral desmosomes (bottom); maximum Z-projection (4-6 μm range). Scale bars: 5 μm. Statistical analyses were performed using the Mann–Whitney U test (B), (D) and (F). Significance is denoted as: ****$p < 0.0001$, ***$p < 0.0005$, ** $p < 0.005$, ns: non-significant. ND: Non-distended, D: Distended. *: goblet cell.

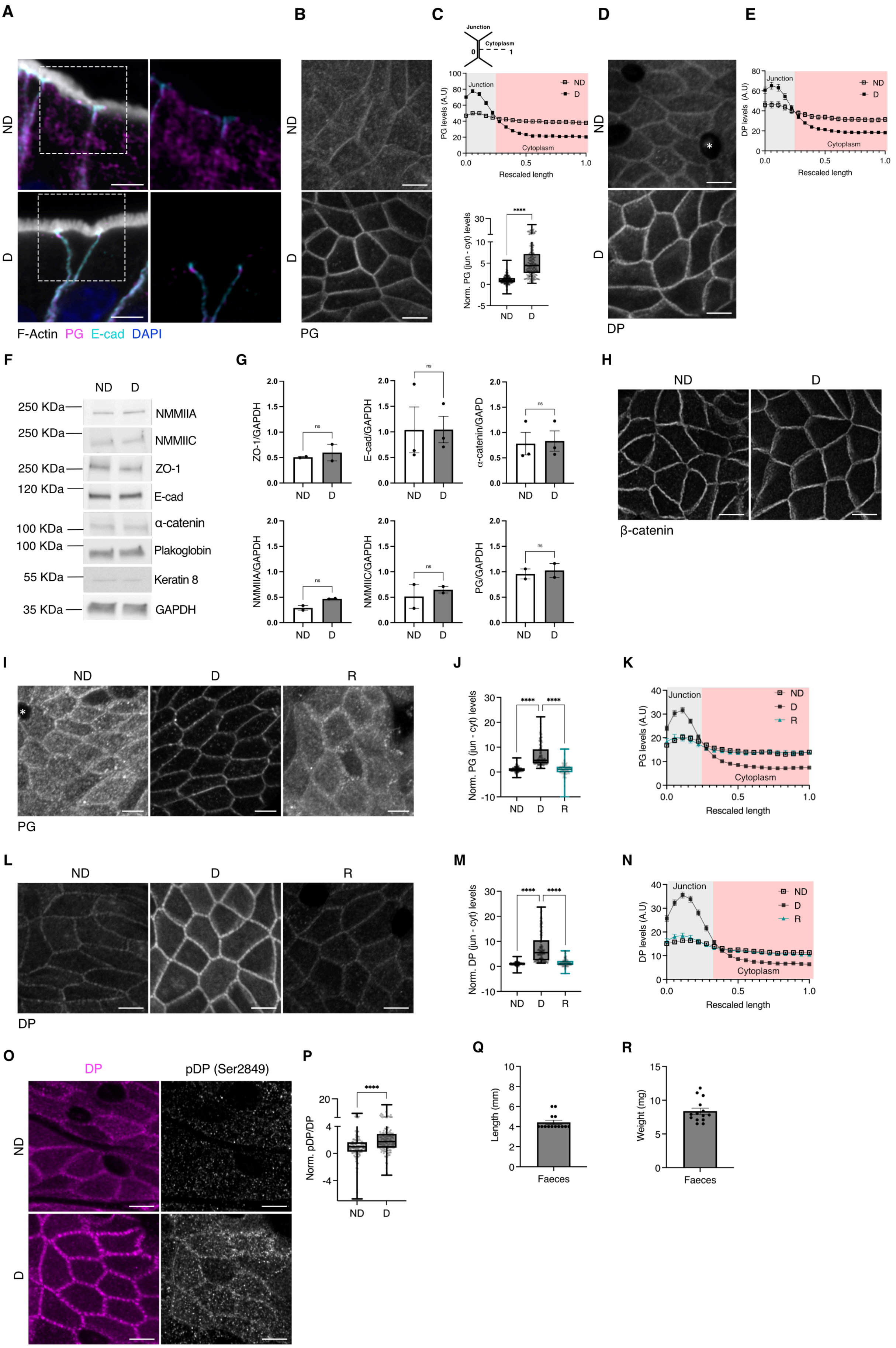

Fig. S2. **(A)** Representative transverse images of colonic epithelium, stained for F-actin (grey), PG (magenta), E-cad (cyan) and

DAPI (blue) in both ND and D regions. Maximum Z-projections (2-4 μm range). Dashed rectangle indicates region shown at higher magnification. Scale bars: 5 μm. **(B)** Representative *en face* images of colonic epithelium, stained for PG in ND and D regions. Maximum Z-projections (1-3 μm range). Scale bar: 5 μm. **(C)** Top: Scheme showing plot profile measurement, briefly a line of fixed length is drawn from junction to cytoplasm and intensity is measured along the line. Middle: Plot profile for PG in ND and D regions of colonic epithelium. Bottom: Box plot showing junctional intensity for PG in ND and D regions (n=28-54 junctions/condition, N=7 independent experiments). **(D)** Representative *en face* images of colonic epithelium, stained for DP in ND and D regions. Maximum Z-projections, 1-3 μm. Scale bar: 5 μm. **(E)** Plot profile for DP in ND and D regions of colonic epithelium. A line of fixed length is drawn from junction to cytoplasm and intensity is measured along the line. **(F-G)** Representative (F) western blot images and (G) quantification for NMMIIA, NMMIIC, ZO-1, E-cad, α-catenin, PG, K8 and GAPDH from protein lysates from ND or D regions of the colonic epithelium. GAPDH was used as a loading control. N=2 independent experiments. **(H)** Representative *en face* images of colonic epithelium stained for β-catenin, showing ND, D regions. Maximum Z-projections (1-3 μm range). Scale bar: 5 μm. **(I)** Representative *en face* images of colonic epithelium stained for PG, showing ND, D and R regions. Maximum Z-projections, 1-3 μm. Scale bar: 5 μm. **(J-K)** Box plot showing: (J) junctional PG intensity in ND, D and R regions (n=44-54 junctions/condition, N=3 independent experiments). (K) Plot profile for PG in ND, D and R regions of colonic epithelium. **(L)** Representative *en face* images of colonic epithelium stained for DP, showing ND, D and R regions. Maximum Z-projections (1-3 μm range). Scale bar: 5 μm. **(M-N)** Box plot showing: (M) junctional DP intensity in ND, D and R regions (n=48-63 junctions/condition, N=3 independent experiments). (N) Plot profile for DP in ND, D and R regions of colonic epithelium. **(O)** Representative *en face* images of colonic epithelium, stained for DP (magenta) and pDP (Ser2849) (grey) in both ND and D regions. Maximum Z-projections (1-3 μm range)**.** Scale bar: 5 μm. **(P)** Box plots showing ratio of junctional intensity (pDP/DP) in ND and D regions (n=49-64 junctions/condition, N=2 independent experiments). **(Q-R)** Bar plots showing length of the faeces pellet(C) and weight of the faeces pellet (D). (n=15 faeces pellets; N=5 independent experiments). Statistical analyses were performed using the Mann–Whitney U test (C), (G) and (P) or Kruskal-Wallis test followed by Dunn's multiple comparison (J) and (M). Significance is denoted as: ****p < 0.0001, ***p < 0.0005, ** p < 0.005, ns: non-significant. ND: Non-distended, D: Distended, R: Relaxed. *: goblet cell.

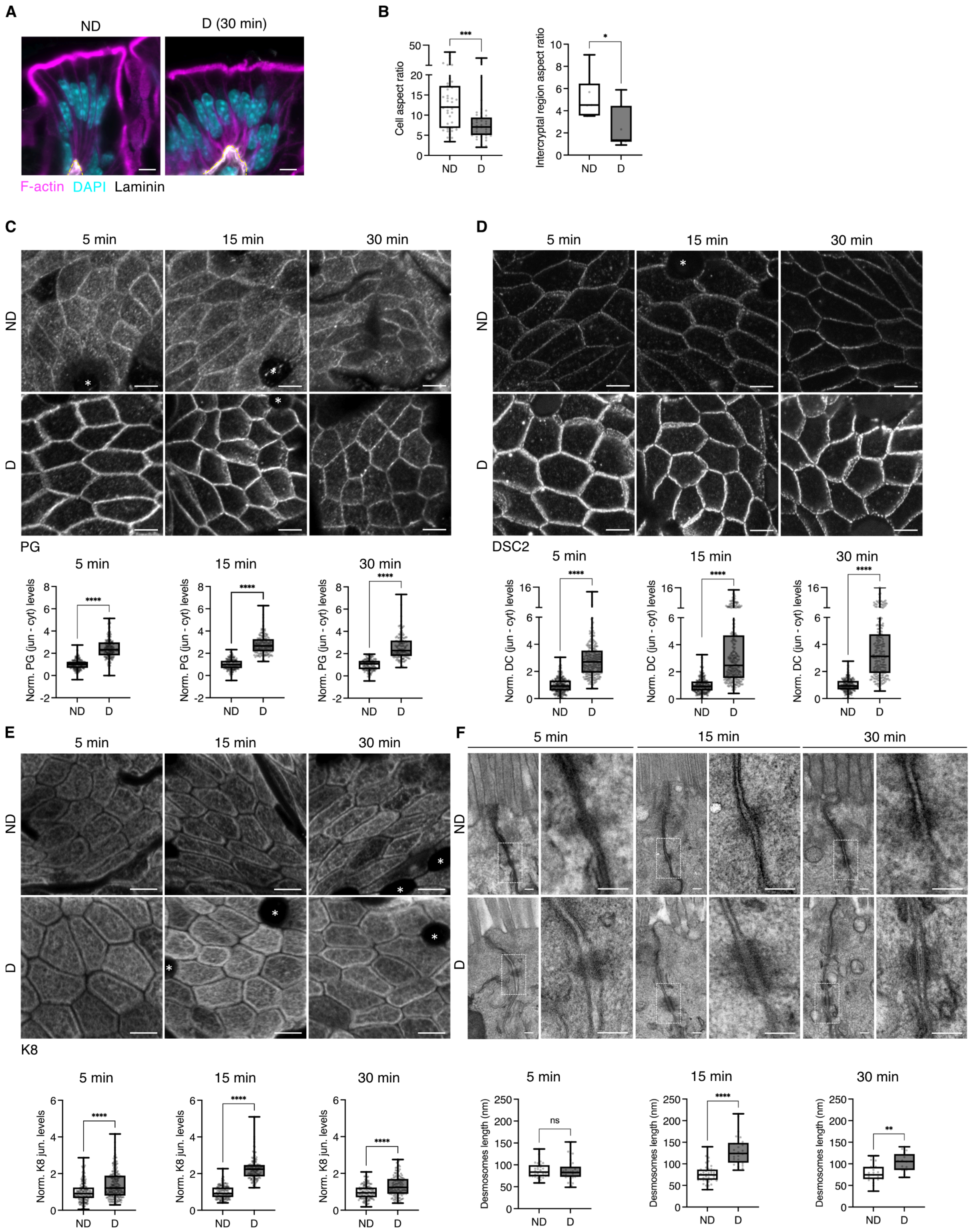

Fig. S3 (A) Representative transverse images of plateau regions in the colonic epithelium, stained for F-actin (magenta), DAPI (cyan) and laminin (grey) in both non-distended (ND) and catheter-mediated distended (D) regions. Maximum Z-projections (2-4 μm range). Scale bar: 5 μm. **(B)** Box plots showing: cell aspect ratio (left; n=15-24 cells/condition) and intercryptal aspect ratio (right; n=3-4 intercryptal regions/condition) in ND and D plateau regions. N=2 independent experiments. **(C)** Top: Representative *en face* images of colonic epithelium, stained for PG in ND and D regions after 5,15 and 30 min of distension. Maximum Z-projections (1-3 μm range). Scale bar: 5 μm. Bottom (left to right): Box plots showing junctional PG intensity in ND and D regions after 5, 15 and 30 min of distension (n=42-57 junctions/condition, N=3-5 independent experiments). **(D)** Top: Representative *en face* images of colonic epithelium, stained for DSC2 in ND and D regions after 5,15 and 30 min of distension. Maximum Z-projection (1-3 μm range). Scale bar: 5 μm. Bottom (left to right): Box plots showing junctional DSC2 intensity in ND and D regions after 5, 15 and 30 min of distension (n=28-61 junctions/condition, N=4 independent experiments). **(E)** Top: Representative *en*

face images of colonic epithelium, stained for K8 in ND and D regions after 5,15 and 30 min of distension. Maximum Z-projection (1-3 μm range). Scale bar: 5 μm. Bottom (left to right): Box plots showing junctional K8 intensity in ND and D regions after 5, 15 and 30 min of distension (n=28-84 junctions/condition, N=4-3 independent experiments). **(F)** Top; Representative transverse TEM images showing colonocyte desmosomes (electron-dense regions, white box; enlarged insets at right side) at the apical side, in ND and D regions after 5, 15 and 30 min of distension. Scale bar: 100 nm. Bottom (left to right): Box plots showing desmosome length in ND and D regions after 5, 15 and 30 min of distension. (n=6-22 desmosome/condition, N=2 independent experiments).

Statistical analyses were performed using the Mann–Whitney U test (B, cell aspect ratio), (C-F) or welch-t-test (B, intercryptal aspect ratio) and F (15 min and 30 min). Significance is denoted as: ****$p < 0.0001$, ***$p < 0.0005$, ** $p < 0.005$, ns: non-significant. ND: Non-distended, D: Catheter-mediated distended. *: goblet cell.

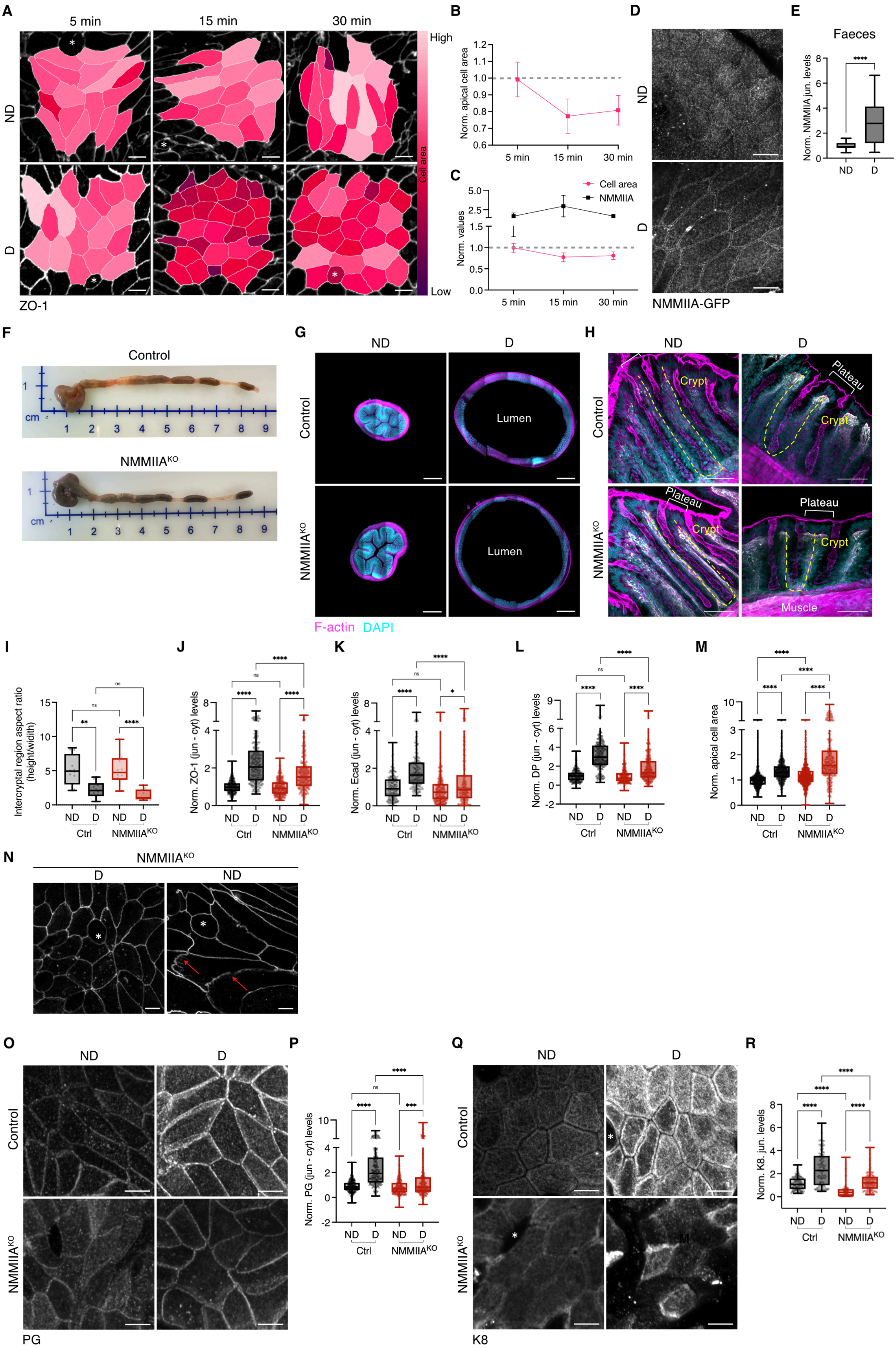

A
5 min
15 min
30 min
High
Low
Cell area
ND
D
ZO-1
B
Norm. apical cell area
5 min
15 min
30 min
C
Cell area
NMMIIA
Norm. values
D
ND
D
NMMIIA-GFP
E
Faeces
Norm. NMMIIA jun. levels
ND
D
F
Control
NMMIIA^KO
cm
G
ND
D
Control
NMMIIA^KO
Lumen
F-actin DAPI
H
ND
D
Control
NMMIIA^KO
Crypt
Plateau
Muscle
I
Intercryptal region aspect ratio (height/width)
J
Norm. ZO-1 (jun - cyt) levels
K
Norm. Ecad (jun - cyt) levels
L
Norm. DP (jun - cyt) levels
M
Norm. apical cell area
ND
D
Ctrl
NMMIIA^KO
N
NMMIIA^KO
D
ND
O
ND
D
Control
NMMIIA^KO
PG
P
Norm. PG (jun - cyt) levels
Q
ND
D
Control
NMMIIA^KO
K8
R
Norm. K8. jun. levels

Fig. S4. (A) Representative images, segmented and colour-coded based on apical cell area in ND and D regions after 5,15 and 30 min of distension. Maximum Z-projections (1-3 μm range). Scale bar: 5 μm. **(B)** Line plot showing mean fold change of apical cell area after 5, 15 and 30 min of distension. Dotted line represents the normalised average for ND. **(C)** Line plot showing mean fold change in cell area and NMMIIA-GFP intensity after 5, 15 and 30 min of distension. Dotted line represents the normalised average for ND. **(D)** Representative *en face* images of colonic epithelium showing NMMIIA-GFP in ND and faeces-D regions. Maximum Z-projection (1-3 μm range). Scale bar: 5 μm. **(E)** Box plot showing junctional NMMIIA-GFP intensity in ND and faeces-D regions (n=18-32 junctions/condition, N=4 independent experiments). **(F)** Representative images of mouse colon, aligned from the proximal (caecum) to the distal (rectum) end, in control and NMMIIA^{KO} mice. **(G)** Representative images of thick section of mouse colonic tissue in transverse view, showing mucosal folds, stained for F-actin (magenta) and DAPI (cyan) in control and NMMIIA^{KO} from ND and faeces-D regions. The panel is a tiled confocal reconstruction. Scale bars: 500 μm. (H) Representative images of thick section of mouse colonic tissue in transverse view, showing, plateau regions (white brackets), muscle layers and crypts (outlined in yellow dashed lines), and stained for F-actin (magenta), DAPI (cyan) and laminin (grey) in control and NMMIIA^{KO} from ND and faeces-D regions. Scale bars: 50 μm. (I) Box plots showing intercryptal aspect ratio in control and NMMIIA^{KO} from ND and faeces-D regions (n= >7 intercryptal regions/condition, N=2 independent experiments). **(J-L)** Box plots showing junctional intensity levels in ND and D regions for ZO-1 (J), E-cad (K), and DP (L) in control and NMMIIA^{KO} (n=38-84 junctions/condition, N=4-5 independent experiments). **(M)** Box plot showing apical cell area in control and NMMIIA^{KO} mice normalized to control ND values (n=42-544 cells/condition, N=3 independent experiments) **(N)** Representative images of NMMIIA^{KO} colonic epithelium stained for ZO-1 in ND and faeces-D regions. Red arrows represent discontinuous ZO-1 staining. Maximum Z-projection (1-3 μm range). Scale bar: 5 μm. **(O)** Representative images of colonic epithelium stained for PG in control and NMMIIA^{KO} from ND and faeces-D regions. Maximum Z-projection (1-3 μm range). Scale bar: 5 μm. **(P)** Box plots showing junctional intensity levels for PG in control and NMMIIA^{KO}, normalized to ND control values (n=60-77 junctions/condition, N=5 independent experiments). **(Q)** Representative images of colonic epithelium stained for K8 in control and NMMIIA^{KO} from ND and faeces-D regions. Maximum Z-projection (1-3 μm range). Scale bar: 5 μm. **(R)** Box plots showing junctional intensity levels for K8 in control and NMMIIA^{KO}, normalized to ND control values (n=45-54 junctions/condition, N=3 independent experiments). Statistical analyses were performed using the Mann–Whitney U test (E) or Kruskal-Wallis test followed by Dunn's multiple comparison (I-M), (P) and (R). Significance is denoted as: ****$p < 0.0001$, ***$p < 0.0005$, ** $p < 0.005$, ns: non-significant. ND: Non-distended, D: Distended. *: Indicates goblet cell.

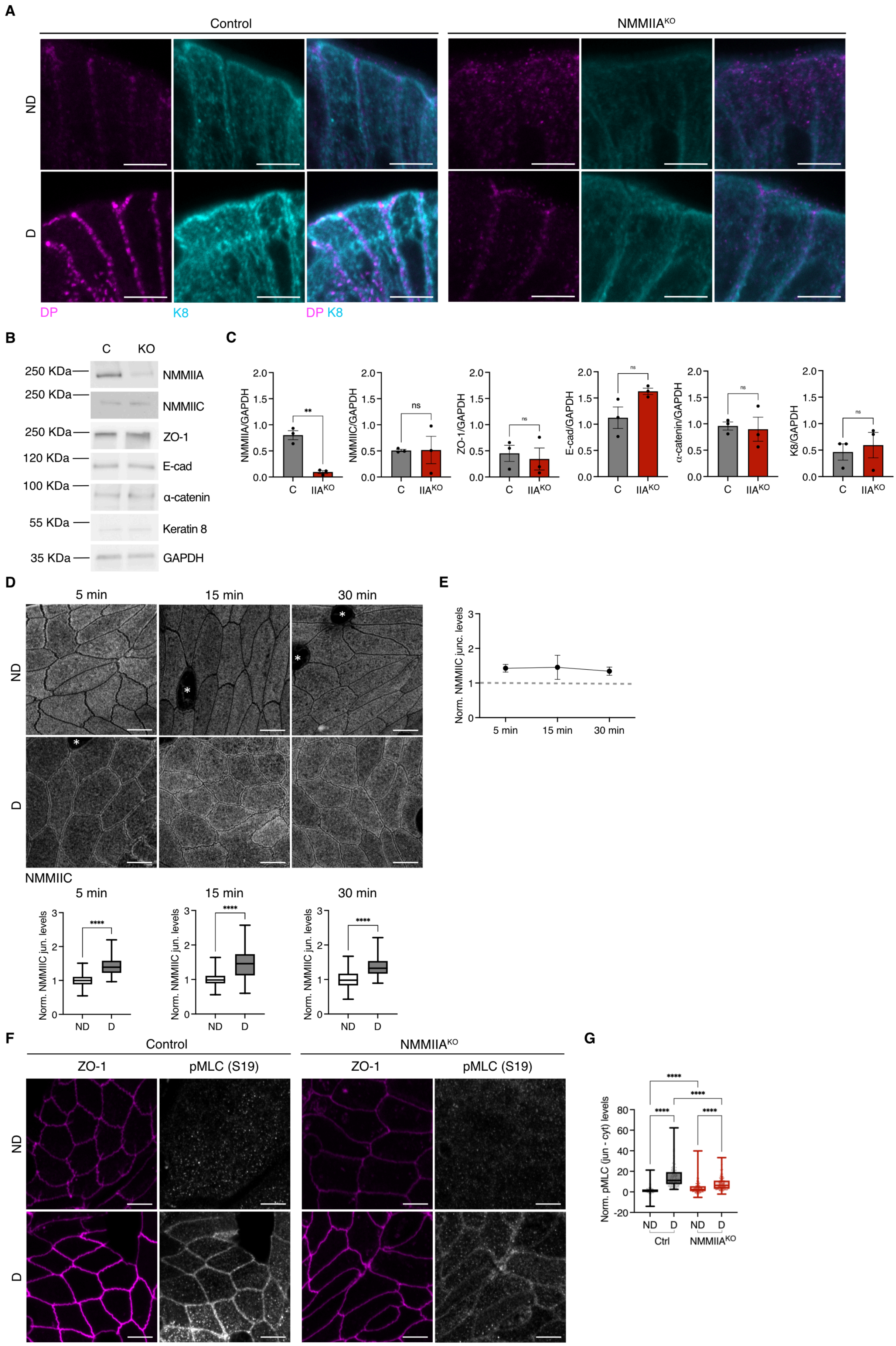

A
Control
NMMIIA^KO
ND
D
DP
K8
DP K8
B
C KO
250 KDa
NMMIIA
250 KDa
NMMIIC
250 KDa
ZO-1
120 KDa
E-cad
100 KDa
α-catenin
55 KDa
Keratin 8
35 KDa
GAPDH
C
NMMIIA/GAPDH
NMMIIC/GAPDH
ZO-1/GAPDH
E-cad/GAPDH
α-catenin/GAPDH
K8/GAPDH
C
IIA^KO
**
ns
D
5 min
15 min
30 min
ND
D
NMMIIC
Norm. NMMIIC jun. levels
****
E
Norm. NMMIIC junc. levels
5 min
15 min
30 min
F
Control
NMMIIA^KO
ZO-1
pMLC (S19)
ND
D
G
Norm. pMLC (jun - cyt) levels
****
ND
D
Ctrl
NMMIIA^KO

Fig. S5. (A) Representative transverse images of colonic epithelium stained for K8 (cyan) and DP (magenta) in control and NMMIIA KO mice from ND and faeces-D regions show strong apical DP and K8 staining, with weaker punctate staining along the basolateral membrane. Maximum Z-projections (2-4 μm range). Scale bar: 5 μm. **(B)** Representative western blot images and **(C)** quantification for NMMIIA, NMMIIC, ZO-1, E-cad, α-catenin, K8 and GAPDH from protein lysates isolated from ND and faeces-D regions of control (C) and NMMIIA^{KO} (KO) mice. GAPDH was used as a loading control (N=3 independent experiments). **(C)** Top: Representative *en face* images of colonic epithelium, stained for NMMIIC in non-distended (ND) and catheter-distended (D) regions after 5, 15 and 30 min of distension. Maximum Z-projection (1-3 μm range). Bottom (left to right): Box plots showing junctional NMMIIC intensity in ND and D regions after 5, 15 and 30 min of distension (n=48-64 junctions/condition, N=2-3 independent experiments). **(E)** Line plot showing mean fold change in NMMIIC intensity after 5, 15 and 30 min of distension. Dotted line represents the normalised average for ND. (F) Representative images of colonic epithelium stained for ZO-1 and pMLC (S19) in control and NMMIIA^{KO} mice from ND and faeces-D regions. Maximum Z-projection (1-3 μm range). Scale bar: 5 μm. (G) Box plots showing junctional intensity levels in ND and D regions for pMLC (S19) in control and NMMIIA^{KO} (n=28-54 junctions/condition, N=3 independent experiments). Statistical analyses were performed using the Welsch-t test (C), Mann-Whitney U test (D) or Kruskal-Wallis test followed by Dunn's multiple comparison (G). Significance is denoted as: ****$p < 0.0001$, ***$p < 0.0005$, ** $p < 0.005$, ns: non-significant. ND: Non-distended, D: Distended. *: goblet cell. Scale bar: 5 μm.

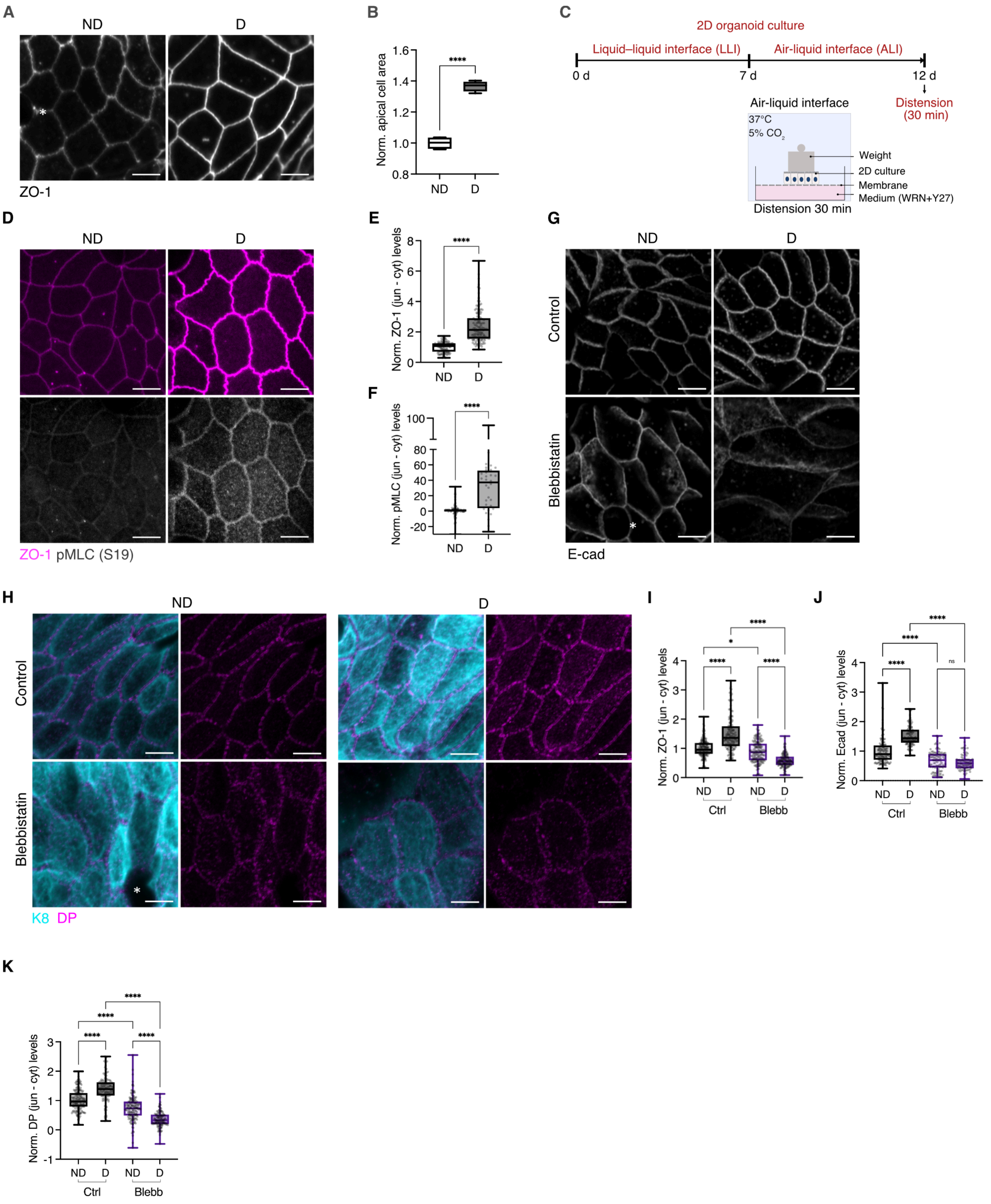

Fig. S6. (A) Representative images showing colonic explants stained for ZO-1 in ND and D after 30 min of distension. Maximum Z-projection (1-3 μm range). Scale bar: 5 μm. **(B)** Box plot showing apical cell area in D-colon explants, normalized to ND regions (n=22-37 cells/condition, N=4 independent experiments). **(C)** Scheme depicting experimental approaches for 2D colonoid culture and distension. **(D)** Representative images of 2D colonoids stained for ZO-1 and pMLC (S19) in ND and D regions. Maximum Z-projection (1-3 μm range). Scale bar: 5 μm. **(E-F)** Box plots showing junctional intensity for ZO-1 (E), and pMLC (F) in ND and D 2D colonoids (n=42-55 junctions/condition, N=2 independent experiments. **(G-H)** Representative images showing colonic explants stained for E-cad (G), and K8 (cyan) and DP (magenta) (H) in ND and D explants after 1 h pre-treatment with blebbistatin or vehicle control (DMSO) followed by 30 min distension. Maximum Z-projection (1-3 μm range). Scale bar: 5 μm. **(I-K)** Box plots showing junctional intensities for ZO-1 (I), E-cad (J) and DP (K) in ND and D explants after pre-treatment with blebbistatin or Vehicle control (DMSO) and distension (n=30-79 junctions/condition, N=2-3 independent experiments). Statistical analyses were performed using the Welch t-test (B), Mann-Whitney U test (E-F) or Kruskal-Wallis test followed by Dunn's multiple comparison (I-K). Significance is denoted as: ****$p < 0.0001$, ***$p < 0.0005$, ** $p < 0.005$, * $p < 0.05$, ns: non-significant. ND: Non-distended, D: Distended. *: goblet cell.

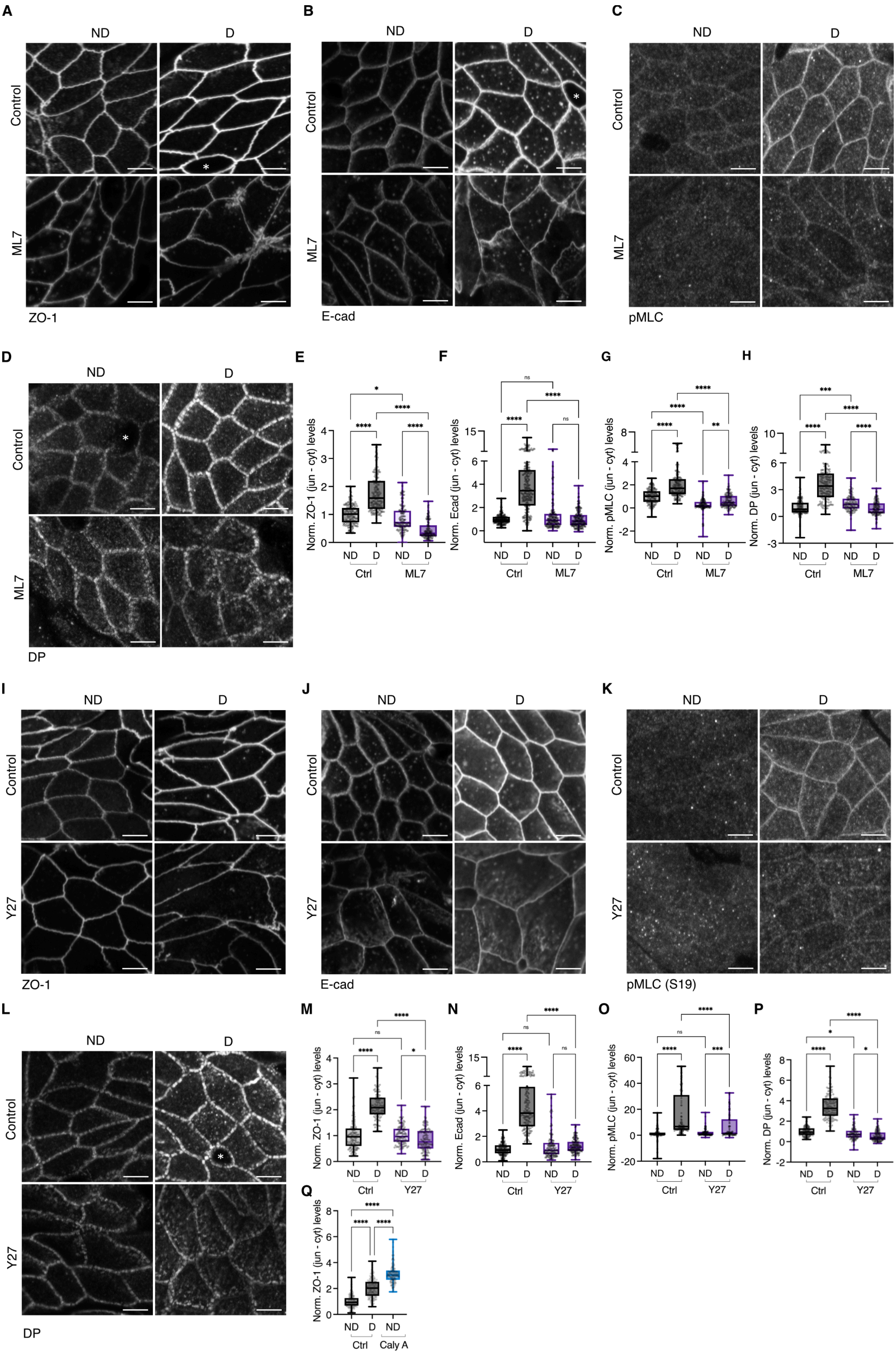

Fig. S7. **(A-D)** Representative images showing colonic explants stained for ZO-1 (A), E-cad (B), pMLC (S19) (C) and DP (D) in

ND and D explants after 1 h pre-treatment with ML-7 or vehicle control (DMSO) followed by 30 min distension. Maximum Z-projection (1-3 μm range). Scale bar: 5 μm. **(E-H)** Box plots showing junctional intensity for ZO-1 (E), E-cad (F), pMLC (G) and DP (H) in ND and D explants after pre-treatment with ML-7 or vehicle control (DMSO) and distension (n=39-74 junctions/condition, N=3-5 independent experiments). **(I-L)** Representative images showing colonic explants stained for ZO-1 (I), E-cad (J), pMLC (S19) (K) and DP (L) in ND and D regions after 1 h pre-treatment with Y27632 or vehicle control (water), followed by 30 min distension. Maximum apical Z-projection (1-3 μm range). Scale bar: 5 μm. **(M-P)** Box plots showing junctional intensity for ZO-1(M), E-cad (N), pMLC (S19) (O) and DP (P) in ND and D explants after pre-treatment with Y27632 (Y27) or vehicle control (water) and distension (n=31-54 junctions/condition, N=3-4 experiments). **(Q)** Box plot showing junctional ZO-1 intensity in Calyculin A-treated ND explants and vehicle-treated ND and D explants (n=53-59 junctions/condition, N=3 independent experiments). Statistical analyses were performed using Kruskal-Wallis test followed by Dunn's multiple comparison (E-H) and (M-Q). Significance is denoted as: ****$p < 0.0001$, ***$p < 0.0005$, ** $p < 0.005$, * $p < 0.05$, ns: non-significant. ND: Non-distended, D: Distended. *: goblet cell.

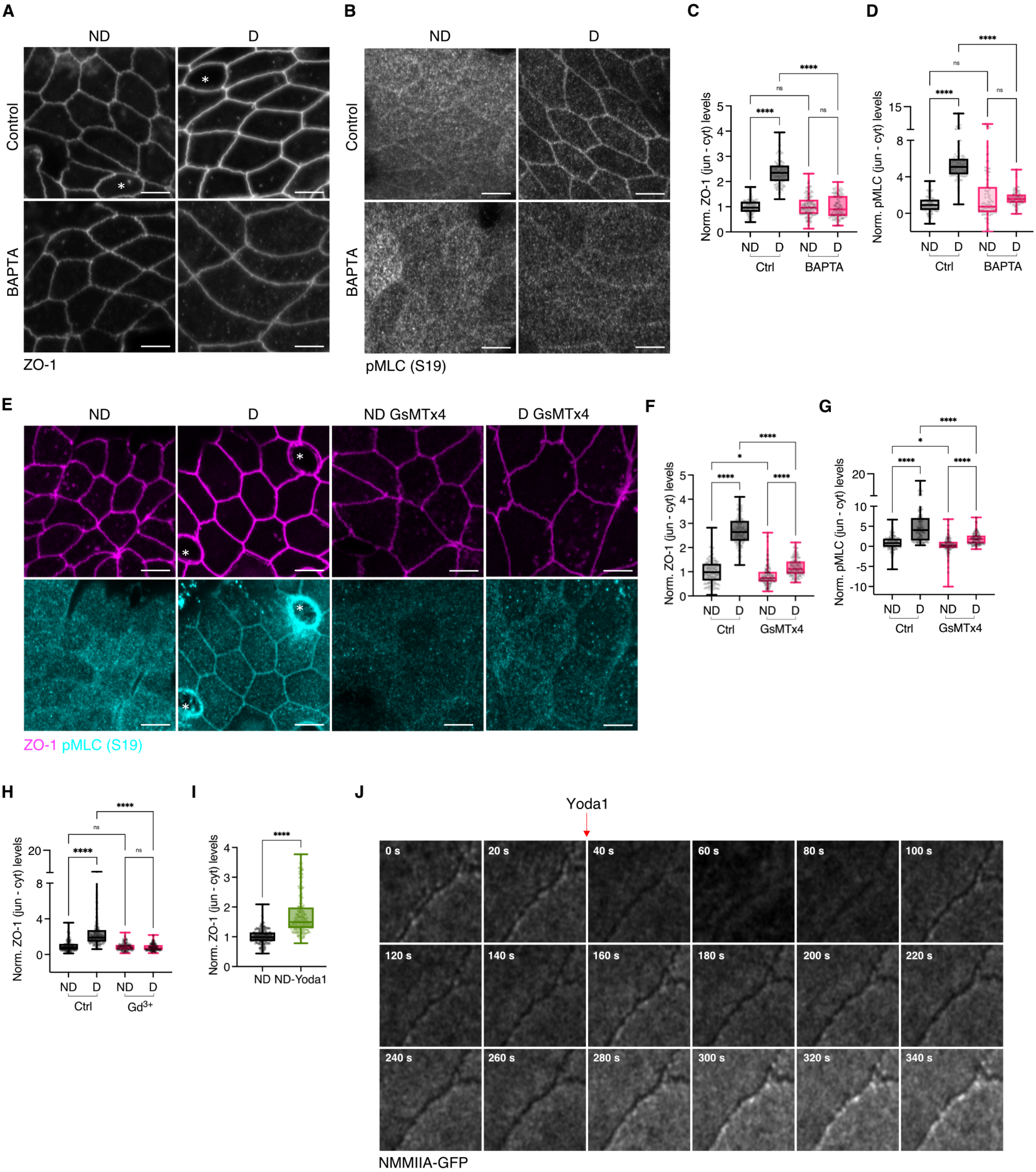

Fig. S8. **(A-B)** Representative image showing colonic explants stained for ZO-1 (A) and pMLC (S19) (B) in ND and D after treatment with BAPTA or vehicle control (water), followed by 30 min distension. Maximum Z-projection (1-3 μm range). Scale bar: 5 μm. **(C-D)** Box plots showing junctional intensity for ZO-1 (C) and pMLC (S19) (D) in ND and D explants after treatment with BAPTA or vehicle control (water) and distension (n=47-54 junctions/condition, N=2 independent experiments). **(E)** Representative image showing colonic explants stained for ZO-1 (magenta) and pMLC (S19) (cyan) in ND and D after treatment with GsMTx-4 or vehicle control (water), followed by 30 min distension. Maximum Z-projection (1-3 μm range). Scale bar: 5 μm. **(F-G)** Box plots showing junctional intensity for ZO-1 (F) and pMLC (S19) (G) in ND and D explants after treatment with GsMTx-4 or vehicle control (water) and distension (n=51-60 junctions/condition, N=3 independent experiments. **(H)** Box plot showing junctional ZO-1 intensity in ND and D explants after pre-treatment with Gd^{3+} or vehicle control (water) and distension (n=58-61 junctions/condition, N=5 independent experiments). **(I)** Box plot showing junctional ZO-1 intensity in ND explants after treatment with Yoda1 and vehicle control (DMSO) (n=55-66 junctions/condition, N=2 independent experiments). **(J)** Representative montage showing junctional recruitment of NMMIIA after addition of Piezo-1 activator, Yoda1. Statistical analyses were performed using the Mann-Whitney U test (I) or Kruskal-Wallis test followed by Dunn's multiple comparison (C-D) and (F-H). Significance is denoted as: ****$p < 0.0001$, ***$p < 0.0005$, ** $p < 0.005$, ns: non-significant. ND: Non-distended, D: Distended. *: goblet cell.

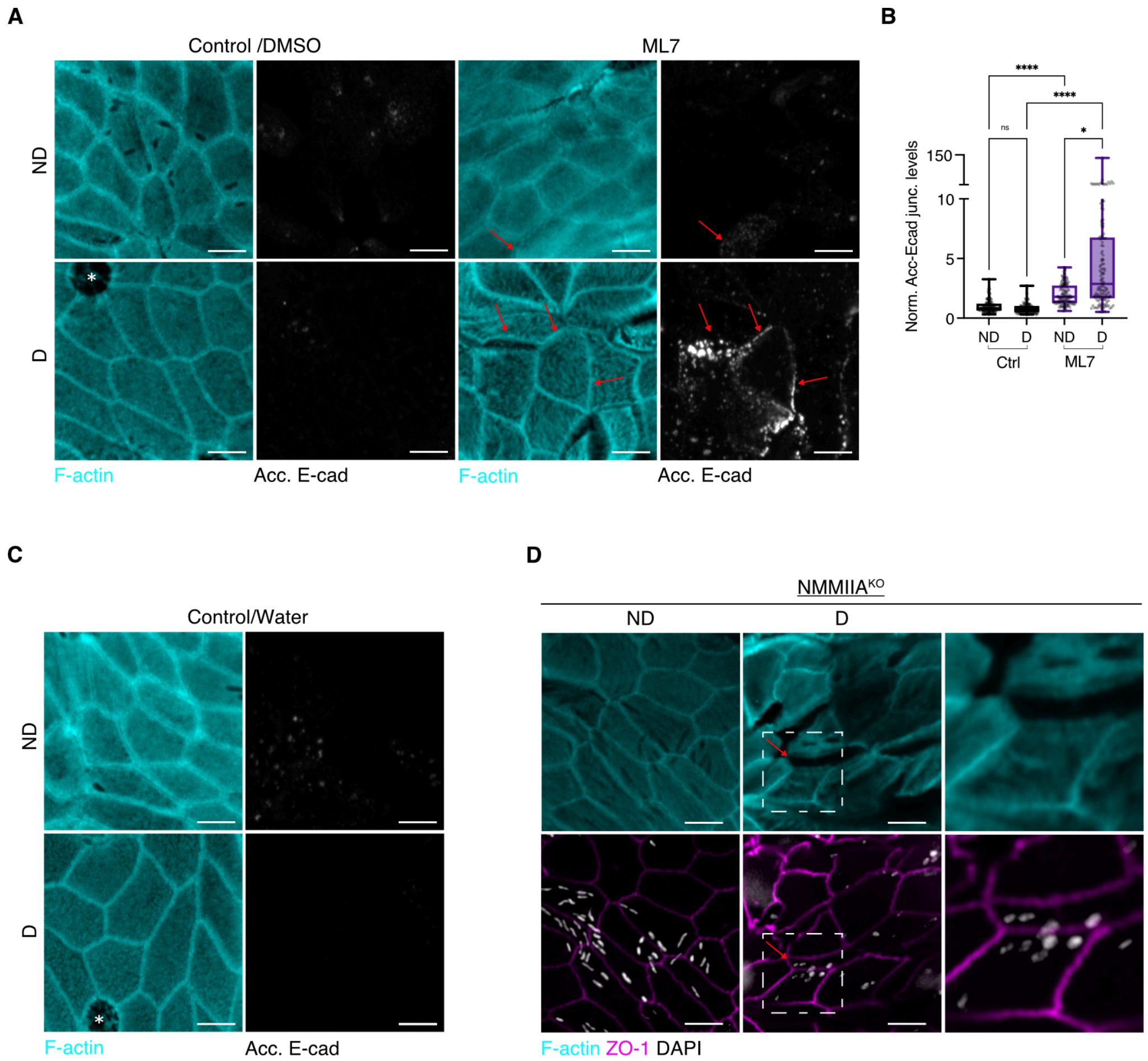

Fig. S9. (A) Representative images showing colonic explants stained for F-actin (cyan) and acc. E-cad (grey) in ND and D after 1 h pre-treatment with ML7 or vehicle (DMSO) followed by 30 min distension. Red arrows indicate breached junctions. Maximum apical Z-projection (1-3 μm range). *: goblet cell. Scale bar 5 μm. **(B)** Box plot showing junctional acc. E-cad intensity in ND and D explants after pre-treatment with ML7 or vehicle control (DMSO) and distension (n=34-69 junctions/condition, N=3 independent experiments). **(C)** Representative image showing colonic explants stained for F-actin (cyan) and acc. E-cad (grey) in ND and D after 1 h pre-treatment with vehicle (water) followed by 30 min distension. Maximum Z-projection (1-3 μm range). Scale bar: 5 μm. **(D)** Representative image of colonic epithelium stained for F-actin (cyan), ZO-1 (magenta) and DAPI (grey) in NMMIIA^{KO} mice from ND and faeces-D regions. Red arrows indicate translocation of bacteria inside the fractured regions. Dashed rectangle indicates region shown at higher magnification. Maximum Z-projection (1-3 μm range). Scale bar: 5 μm. Statistical analyses were performed using Kruskal-Wallis test followed by Dunn's multiple comparison (B). Significance is denoted as: ****p < 0.0001, ***p < 0.0005, ** p < 0.005, ns: non-significant. ND: Non-distended, D: Distended. *: goblet cell.
